# Immunostimulatory Hydrogel with Synergistic Blockage of Glutamine Metabolism and Chemodynamic Therapy for Postoperative Management of Glioblastoma

**DOI:** 10.1002/advs.202412507

**Published:** 2025-02-20

**Authors:** Yiran Guo, Tianhe Jiang, Sen Liang, Anhe Wang, Jieling Li, Yi Jia, Qi Li, Jian Yin, Shuo Bai, Junbai Li

**Affiliations:** ^1^ State Key Laboratory of Biopharmaceutical Preparation and Delivery Institute of Process Engineering Chinese Academy of Sciences Beijing 100190 China; ^2^ Key Laboratory of Carbohydrate Chemistry and Biotechnology Ministry of Education School of Biotechnology Jiangnan University Wuxi Jiangsu 214122 China; ^3^ University of Chinese Academy of Sciences Beijing 100049 China; ^4^ Beijing National Laboratory for Molecular Sciences (BNLMS), CAS Key Lab of Colloid, Interface and Chemical Thermodynamics Institute of Chemistry Chinese Academy of Sciences Beijing 100190 China

**Keywords:** glioblastoma multiforme, glutamine metabolism, self‐assembly, short peptides

## Abstract

Glioblastoma multiforme (GBM) is one of the most lethal malignant brain tumors in the central nervous system. Patients face many challenges after surgery, including tumor recurrence, intracranial pressure increase due to cavitation, and limitations associated with immediate postoperative oral chemotherapy. Here an injected peptide gel with in situ immunostimulatory functions is developed to coordinate the regulation of glutamine metabolism and chemodynamic therapy for overcoming these postoperative obstacles. The methodology entails crafting injectable gel scaffolds with short peptide molecules, incorporating the glutaminase inhibitor CB‐839 and copper peptide self‐assembled particles (Cu‐His NPs) renowned for their chemodynamic therapy (CDT) efficacy. By fine‐tuning glutamic acid production via metabolic pathways, this system not only heightens the therapeutic prowess of copper peptide particles in CDT but also escalates intracellular oxidative stress. This dual mechanism culminates in augmented immunogenic cell death within glioblastoma multiforme cells and improves a conducive immune microenvironment. Based on the concept of metabolic reprogramming, this treatment strategy has great potential to significantly reduce GBM tumor recurrence and prolong median survival in murine models.

## Graphical abstract

To address the challenge of postoperative recurrence, Combo Gel, a peptide‐based hydrogel that integrates glutamine metabolism modulation with chemodynamic therapy (CDT) is engineered. This innovative system, containing CB‐839 and copper peptide nanoparticles (Cu‐His NPs), enhances CDT's efficacy and promotes immunogenic cell death, thereby strengthening the immune response against GBM.

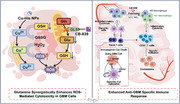

## 1 Introduction

Glioblastoma multiforme (GBM) is the most aggressive and lethal malignant brain tumor within the central nervous system.^[^ ^1^ ^]^ Standard clinical management typically involves surgical resection, followed by adjuvant chemotherapy and radiotherapy.^[^ ^2^ ^]^ However, the infiltrative nature of GBM often prevents complete surgical removal, and surgery can disrupt the tumor's immune microenvironment, potentially delaying chemotherapy and leading to tumor recurrence.^[^ ^3^ ^]^ Additionally, the post‐surgical cavity provides space for GBM recurrence.^[^ ^1^ ^]^ The blood–brain barrier (BBB) further complicates the delivery of drugs and immune cells to the brain.^[^ ^4^ ^]^ The malignant growth characteristics of GBM, coupled with the unique postoperative treatment needs, pose significant challenges for drug delivery methods and therapeutic strategies following GBM surgery.^[^ ^5^ ^]^

Metabolism plays a pivotal role in the vitality, expansion, invasiveness, and metastatic potential of tumor cells.^[^ ^6^ ^]^ Unlike normal cells, cancer cells undergo metabolic reprogramming to satisfy their heightened demands for energy and nutrients, inevitably leading to competition for nutrients with coexisting cells.^[^ ^7^ ^]^ Notably, the metabolic reprogramming of tumor cells can facilitate information exchange and crosstalk with immune cells through metabolites, indicating that cellularmetabolic processes should not be considered in isolation.^[^ ^8^ ^]^ Regulating cellular metabolism can influence the functional changes in downstream immune cells and reshape the immune microenvironment, offering a fresh perspective for exploring the interplay between cell metabolism and immunity.^[^ ^9^ ^]^ In particular, glutamine plays a key role in cancer cell metabolism.^[^ ^10^ ^]^ The regulation of glutamine metabolism in cancer cells also affects the communication and crosstalk with immune cells in the microenvironment through metabolites.^[^ ^11^ ^]^ This interaction influences the polarization of tumor‐associated macrophages (TAMs) and enhances the activity of T lymphocytes and cytokine expression by depriving cancer cells of essential nutrients, thereby improving the efficacy of immunotherapy.^[^ ^12^ ^]^ Given the dual significance of suppressing tumor growth and immune modulation, targeting glutamine metabolism has emerged as a key focus for cancer therapy. CB‐839, a small molecule glutaminase inhibitor currently in clinical trials (NCT03047993 and NCT02771626), has shown promise.^[^ ^13^ ^]^ However, clinical outcomes have not met expectations due to tumor heterogeneity, metabolic adaptability, the complex immuno‐suppressive microenvironment, and challenges in drug delivery, underscoring the limitations of monotherapy.^[^ ^14^ ^]^ Research indicates that inhibiting glutamine catabolism can reduce glutamate levels, a precursor to glutathione (GSH), thereby decreasing GSH content. This reduction impairs the tumor cells' ability to clear intracellular reactive oxygen species (ROS),^[^ ^15^ ^]^ establishing a link for synergistic dynamic therapy through glutamine modulation.^[^ ^16^ ^]^ For deeply situated tissues like GBM in the brain, CDT for ROS delivery is ideal.^[^ ^17^ ^]^ Copper‐based nanomaterials, more efficient and with a broader pH tolerance than iron‐based counterparts, are used to generate CDT.^[^ ^18^ ^]^ However, excessive free copper ions may lead to severe systemic toxicity, so it is necessary to limit copper ions in time and space, allowing them to be stable in the physiological environment but rapidly released in tumor cells, producing ROS through the Fenton reaction to kill tumors.^[^ ^18^, ^19^ ^]^ In the unique postoperative treatment scenarios of GBM, drug delivery systems must adhere to rigorous temporal and spatial specifications.^[^ ^20^ ^]^ Injectable peptide hydrogels can fill the irregular cavities left by surgery, physically restricting tumor proliferation, and facilitating a controlled release of therapeutic agents through the modulation of hydrogel degradation.^[^ ^21^ ^]^ Moreover, endogenous short peptides have excellent biocompatibility and low immunogenicity, making them easy to regulate assembly. This allows for the regulation of carrier degradation time and pH‐triggering conditions to meet the complex requirements of sustained drug release.^[^ ^22^ ^]^ Consequently, in this study, we presented a hydrogel constituted via the self‐assembly of Fmoc‐tyrosine‐tyrosine‐lysine (Fmoc‐YYK) and Fmoc‐tyrosine‐aspartate (Fmoc‐YD). The short peptides engaged in molecular self‐assembly driven by electrostatic interactions, ultimately resulting in a fibrous supramolecular hydrogel structure.

**Scheme 1:**
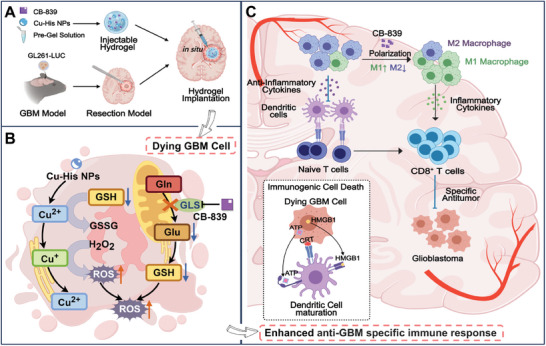
Intracavitary delivery of Combo Gel, an injectable hydrogel formulation, in a mouse model post‐glioblastoma surgery and its mechanism to inhibit GBM relapse. A) The preparation of Combo Gel and its direct in situ injection into the post‐surgical cavity. B) Illustrates the release of Cu‐His NPs and CB‐839 from the hydrogel into the GBM microenvironment, where Cu‐His NPs initiate CDT, and CB‐839 suppresses glutaminase activity, leading to a decrease in GSH and subsequent amplification of oxidative stress, ultimately inducing GBM cell death. C) An abundance of ROS damages tumor cells, inducing ICD. This process releases damage‐associated molecular patterns (DAMPs), which in turn trigger an anti‐tumor immune response. This response promotes the maturation of dendritic cells (DCs) and the activation of T cells. Simultaneously, CB‐839 facilitates the polarization of M2 macrophages toward M1 macrophages, thereby reprogramming the tumor microenvironment.

In this study, we have developed an innovative postoperative treatment system for GBM. Our approach involves the utilization of an in situ injectable peptide hydrogel that is loaded with the glutaminase inhibitor CB‐839 and self‐assembling Cu^2+^‐peptide coordination nanoparticles (Cu‐His NPs) for CDT (**Scheme** **1**A). As depicted in Scheme 1B, CB‐839 effectively suppresses glutamine production, thereby reducing intracellular GSH levels and enhancing the cytotoxicity of Cu^2+^‐mediated CDT. This occurs by diminishing GSH's neutralizing effect on ROS. Moreover, CDT induces a significant increase in ROS production, leading to amplified oxidative stress within tumor cells. Consequently, this triggers immunogenic cell death (ICD) and initiates an immune response to counteract the immunosuppressive tumor microenvironment (Scheme 1C).^[^ ^23^ ^]^ Short peptide molecules, as building blocks of our carriers, show remarkable flexibility and versatility in the assembly process, for instance, to control the mechanical strength and degradation time of the hydrogel.^[^ ^24^ ^]^ Remarkably, our experimental results demonstrate that this immunotherapy strategy, based on metabolic reprogramming, effectively addresses the complex treatment scenarios following GBM surgery. It successfully inhibits GBM recurrence and significantly prolongs overall survival in mice.

## 2 Results and Discussion

### 2.1 Synthesis and Characterization of Combo Gel

**Figure 1:**
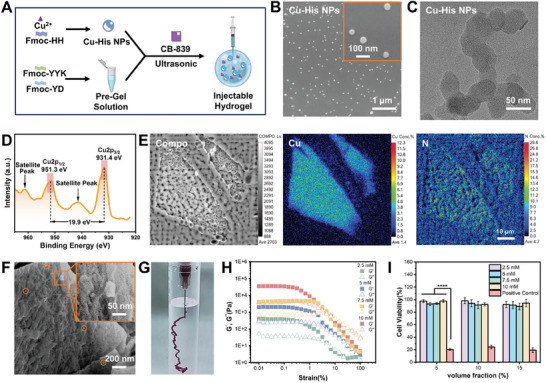
Characterization of the injectable hydrogel Combo Gel. A) Schematic illustration of Combo Gel preparation. B) SEM image of Cu‐His NPs. C) HRTEM image of Cu‐His NPs. D) XRD spectra of Cu‐His NPs and Fmoc‐HH, with black arrows indicating the positions of satellite peaks. E) The COMPO images and elemental distribution images of Cu and N for Cu‐His NPs detected by EPMA. F) SEM image of Combo Gel. G) Combo Gel, when injected through a 22‐gauge needle into water, immediately forms a stable gel. H) Amplitude sweep curves of Fmoc‐YD/YYK hydrogels at various concentrations at 37 °C. Experiments were repeated three times. I) Biocompatibility of Fmoc‐YD/YYK hydrogels at different concentrations compared to a positive control group. Fmoc‐YD/YYK refers to a 1:1 mixture of Fmoc‐YD and Fmoc‐YYK, *n* = 3.

In this study, we successfully synthesized an injectable peptide hydrogel (Combo Gel) loaded with Cu^2+^‐peptide nanoparticle (Cu‐His NP) complexes and the glutaminase inhibitor CB‐839 (**Figure** **1**A). Initially, we selected short peptide molecules with positively charged Fmoc‐Tyrosine‐Tyrosine‐Lysine (Fmoc‐YYK) and negatively charged Fmoc‐Tyrosine‐Aspartic acid (Fmoc‐YD) to prepare injectable hydrogel scaffolds.^[^ ^25^ ^]^ These molecules utilized a variety of intermolecular forces, such as electrostatic and *π–π* interactions, to assemble with flexibility and diversity.^[^ ^21^, ^26^ ^]^ The hydrogel scaffold is semi‐transparent at room temperature (Figure S1A, Supporting Information), and the scanning electron microscope (SEM) image reveals the common fibrous morphology of the hydrogel at the micro‐scale (Figure S1B, Supporting Information). Fourier transform infrared spectroscopy (FT‐IR) analysis revealed absorption peaks at 1690 and 1615 cm^−1^, characteristic of the antiparallel β‐sheet secondary structure in the peptide hydrogel (Figure S2, Supporting Information).^[^ ^27^ ^]^

Cu^2+^‐based CDT nanomaterials have demonstrated promising potential in tumor treatment. However, the targeted release of Cu^2+^ and issues related to free Cu^2+^ have constrained their advancement. In this study, we coordinated histidine, featuring an imidazole group, with Cu^2+^ to develop CDT generators. The histidine, known for its potent membrane‐penetrating ability,^[^ ^28^ ^]^ was used to control the release of Cu^2+^ specifically in the tumor microenvironment. Comparing the SEM image of Cu‐His NPs in Figure 1B, the high‐resolution transmission electron microscopy (HRTEM) image of Cu‐His NPs in Figure 1C, and the HRTEM image of the raw material Fmoc‐Histidine‐Histidine (Fmoc‐HH) in Figure S3 (Supporting Information), it could be observed that the morphology of Cu‐His NPs was distinctly different from that of Fmoc‐HH, forming spherical particles with a diameter of ≈40 nm. HRTEM analysis revealed a lattice spacing of 0.324 nm for the Cu‐His NPs, slightly larger than the 0.225 nm spacing of Fmoc‐HH, confirming the formation of ligand‐bound nanoparticles between Cu^2+^ and Fmoc‐HH (Figure S4, Supporting Information). The FT‐IR spectrum of Cu‐His NPs and Fmoc‐HH revealed that in the presence of Cu^2+^, the stretching vibration of the N─H bond in the histidine imidazole group shifted from 3294 to 3146 cm^−1^, and the absorption peaks for the stretching vibrations of C═O and C─N bonds significantly intensified at 1656 and 1132 cm^−1^, respectively (Figure S5, Supporting Information). The shifts and enhancements of these vibrational peaks indicated that Cu^2+^ had formed coordination bonds with nitrogen or oxygen atoms within the peptide chain. X‐ray photoelectron spectroscopy analysis showed peaks for Cu2p, O1s, N1s, and C1s at 933.1, 529.1, 398.1, and 283.1 eV, respectively (Figure S6, Supporting Information), with the Cu2p_3/2_ and Cu2p_1/2_ peaks located at 931.4 and 951.3 eV. The main peaks were separated by a 19.9 eV spin energy, and satellite peaks were observed at 941 and 961 eV, indicating the presence of Cu^2+^ in the coordination (Figure 1D).^[^ ^29^ ^]^ Electron probe microanalysis (EPMA) further confirmed the uniform distribution of Cu^2+^ throughout the sample, indicating that the Cu‐His NPs were formed by the self‐assembly of Cu^2+^ and Fmoc‐HH (Figure 1E).

The CDT generator Cu‐His NPs and the glutaminase inhibitor CB‐839 were exclusively encapsulated in hydrogel scaffolds. SEM images revealed that the Cu‐His NPs were uniformly dispersed within the hydrogel's fibrous matrix (Figure 1F), and the formulation demonstrated superior injectability (Figure 1G). Rheological testing indicated that Combo Gel's viscosity significantly decreased with an increase in shear rate from 0.1 to 100 s^−1^, showcasing its pronounced shear‐thinning behavior (Figure S7, Supporting Information). Given the unique mechanical characteristics of brain tissue, with a modulus of ≈1–2 kPa, any mechanical discrepancy between implanted materials and the brain could potentially induce epilepsy or other undesirable immune reactions.^[^ ^30^ ^]^ Therefore, gel scaffolds with varying mechanical strengths were fabricated by adjusting the assembly of short peptide molecules. Select the gel scaffold matching the mechanical properties of brain tissue (Figure 1H), and verify the biocompatibility of the gel scaffold(Figure 1I).

### 2.2 In Vitro Anti‐Tumor Studies of Combo Gel

**Figure 2:**
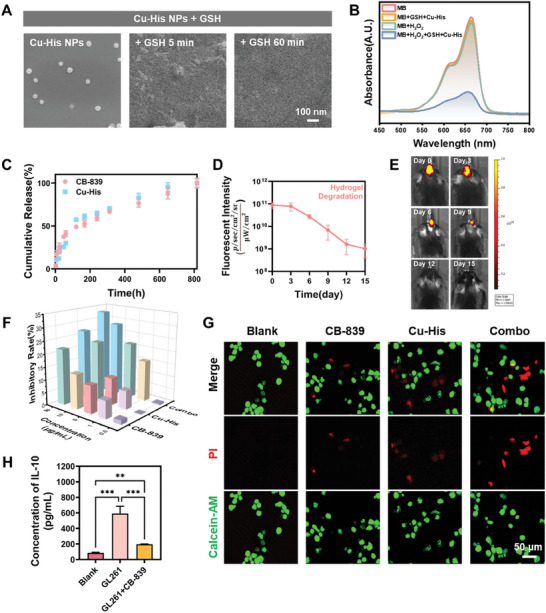
The CDT mechanism of Cu‐His NPs and the synergistic cytotoxic effects of Cu‐His NPs and CB‐839 in vitro, along with the hydrogel's capacity to extend the local retention and release of drugs. A) SEM images of Cu‐His NPs mixed with GSH at various time intervals. B) The degradation of MB after a 4‐hour reaction confirms the Fenton‐like reaction between Cu^+^ and H_2_O_2_, producing ·OH. Experiments were repeated three times. C) The cumulative release profiles of Cu‐His NPs and CB‐839 from the hydrogel, *n* = 3. D) The degradation of the hydrogel in the brains of mice was quantitatively analyzed, *n* = 5. E) Fluorescence images of the hydrogel retention in the brains of mice at specific time points were captured using an IVIS system. The hydrogel was labeled with Cy5 for tracking. *n* = 5. F) The inhibitory effects of Cu‐His NPs and CB‐839 on the GL261 cell line were assessed after 24 h of incubation, *n* = 3. G) Live/dead staining of GL261 cells after 12 h of exposure to Cu‐His NPs and CB‐839, with green fluorescence representing live cells stained with calcein AM and red fluorescence indicating dead cells stained with propidium iodide (PI). H) In vitro immunocrosstalk assay. The supernatants of each fraction were assayed for IL‐10 secretion by macrophages after co‐culture with macrophages, Data are presented as the mean ± S.D, *n* = 6.

Next, we investigated the in vitro antitumor efficacy of Combo Gel. The Cu^2+^ was capable of undergoing a redox reaction with glutathione (GSH), resulting in the formation of Cu^+^ and oxidized glutathione (GSSG). When equal mass ratios of Cu‐His NPs and GSH were combined in an aqueous solution, a rapid emergence of purplish‐red fluorescence was observed under UV light (Figure S8A, Supporting Information), indicating the occurrence of a redox reaction.^[^ ^31^ ^]^ Over time, this fluorescence gradually faded, confirming the progression of the reaction. The fluorescence and emission spectra of the mixtures were further confirmed using a fluorescence spectrophotometer (Figure S8B, Supporting Information). SEM analysis showed a significant morphological change in the Cu‐His NPs after only 5 min of reaction, with the nanoparticles losing their spherical integrity (**Figure** **2**A), suggesting an extremely rapid reaction kinetics. The reaction product in Figure S9A (Supporting Information) exhibits peaks similar to those in Figure S9B (Supporting Information) of the commercialized GSSG, particularly in the amide I band, amide II band, and the disulfide region, leading to the inference that the product in Figure S9A (Supporting Information) is likely GSSG. The peaks in the 500–600 cm^−1^ range of Figure S9A (Supporting Information) may be associated with the stretching vibrations of the disulfide bond (─S─S─), which is a characteristic peak of GSSG, further supporting the formation of GSSG. In the HRMS spectrum of Figure S10 (Supporting Information), the most prominent peak occurs at m/z = 611.14689. This value is likely to be the molecular weight of GSSG. Considering protonation or other charges that may occur during the oxidation process, the measured molecular weight may differ slightly. Coupled with the analysis of the 1H‐NMR in Figure S11 (Supporting Information), which shows the chemical shifts, peak shapes, number of peaks, and peak areas of the product compared to the commercial GSSG, it is essentially confirmed that the product is GSSG.^[^ ^32^ ^]^ These findings confirmed that Cu‐His NPs degraded in the presence of GSH, undergoing a redox reaction to produce Cu^+^ and GSSG. Methylene blue (MB), known for its characteristic UV‐absorption peak, can be degraded by hydroxyl radicals (·OH). Figure 2B illustrates that the UV absorption peaks of MB reacted with H_2_O_2_ alone or with a mixture of Cu‐His NPs and GSH showed minimal changes after 4 h. However, significant changes in the UV absorption peaks were observed when MB, H_2_O_2_, Cu‐His NPs, and GSH were all present, suggesting that the Fenton‐like reaction of Cu^2+^ with H_2_O_2_ in the presence of GSH can initiate a cascade reaction to produce ·OH. The rate of ·OH production in the absence of GSH was significantly lower compared to when GSH was added, as observed after mixing MB, H_2_O_2_, and Cu‐His NPs (Figure S12, Supporting Information). This further supports the notion that Cu‐His NPs can more effectively generate ·OH under GSH influence, targeting tumor cell destruction. Figure S13 (Supporting Information) showed that the rate of ·OH production in the presence of Cu‐His NPs and H_2_O_2_ gradually slowed down as the reaction time increased with GSH.

The intact release of Cu‐His NPs from the hydrogel was investigated at 37 °C (Figure S14, Supporting Information). The results showed that the spherical nanoparticle structure of Cu‐His NPs was maintained after their release from the hydrogel after 12 h. Then, we immersed hydrogels loaded with Cu‐His NPs (Cu‐His Gel) and CB‐839 (CB‐839 Gel) separately in phosphate‐buffered saline (PBS) and incubated at 37 °C. The release concentrations were monitored using high‐performance liquid chromatography. The release profiles revealed that by day 5, the release amounts of Cu‐His NPs and CB‐839 were 57% and 49%, respectively, with the release rate subsequently decreasing. By day 20, ≈80% of both drugs had been released (Figure 2C). Both agents followed zero‐order release kinetics, suggesting that the hydrogel scaffold effectively sustained drug release, extending the retention time in the mouse brain. The drug release rate was found to be nearly concurrent with the hydrogel volume loss, indicating that release was primarily dependent on hydrogel degradation. To further evaluate the in vivo release behavior, Combo Gel was injected into the brains of mice, and the Cy5‐labeled hydrogel signal was quantitatively monitored at set time points using an in vivo imaging system (IVIS) (Figure 2D,E). The injected hydrogel rapidly underwent the solution‐to‐gel transition within the mouse cranium, and this swift in situ gelation effectively prevented rapid drug release. Continuous monitoring indicated that the gel remained in the mice for approximately two weeks, with no significant fluorescent signals detected in other major organs or blood (Figure S15, Supporting Information), and the mice's body weight remained unaffected (Figure S16, Supporting Information). The mice's major organs were harvested for H&E staining, showing no notable differences compared to healthy mice, which suggested that the gel formulation possessed excellent biocompatibility (Figure S17, Supporting Information).

We further investigated the synergistic tumor cell‐killing effects of Cu‐His NPs combined with CB‐839 in vitro. The Cell Counting Kit‐8 (CCK‐8) assay was utilized to assess the cell inhibition rates of the GL261 GBM cell line following 24 h of treatment with Cu‐His NPs, CB‐839 alone, or their combination (Figure 2F). The findings indicated that the cell inhibition rate with CB‐839 and Cu‐His NPs co‐cultured with tumor cells was markedly higher than that of single‐agent treatments. At a Combo group drug concentration of 10 µg mL^−1^, the inhibition rate after 24 h reached 27%, exhibiting strong cytotoxicity. As the drug concentration increased, the cytotoxicity of the combined treatment against cancer cells was also significantly enhanced. Live/dead cell staining, after culturing at a drug concentration of 10 µg mL^−1^ for 12 h, yielded the same conclusion: the synergistic effect of Cu‐His NPs and CB‐839 amplified the cytotoxicity against GL261 glioma cells in vitro, with a significantly higher cell inhibition rate than monotherapy (Figure 2G).

To demonstrate that CB‐839 reprograms glutamine metabolism in tumor cells, thereby causing immunomodulation and reducing the polarization of macrophages to the M2 phenotype, we designed in vitro cell interaction experiments.^[^ ^33^ ^]^ We measured the levels of IL‐10 secreted by RAW264.7 macrophages when co‐cultured with supernatants from tumor cells under various treatment conditions using an ELISA. The results showed that compared to the untreated control group, macrophages co‐cultured with supernatant from the GL261 cell line exhibited a significant increase in IL‐10 secretion, approximately seven times that of the control group. However, when the GL261 cell line was treated with CB‐839, and the supernatant was used to co‐culture macrophages, the induced IL‐10 concentration was significantly lower than that from the untreated GL261 cell line, with IL‐10 levels dropping to ≈195.9 pg mL^−1^, which is about twice that of the control group(Figure 2H).

**Figure 3:**
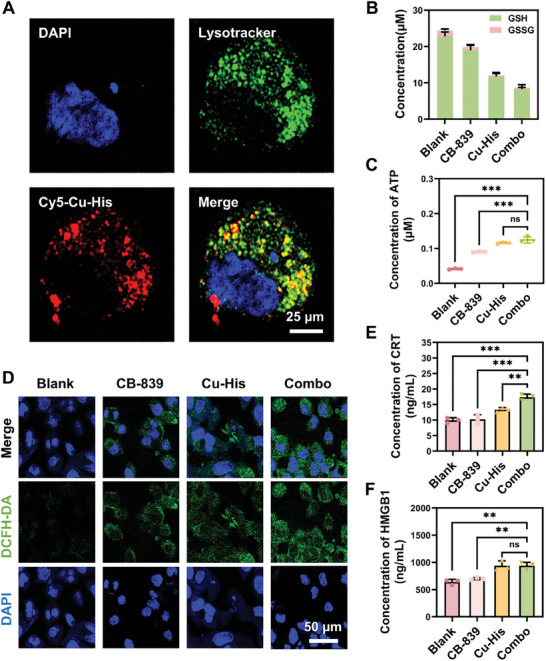
Cellular uptake of Cu‐His NPs and the augmentation of ROS generation and ICD effects through synergistic therapy. A) Confocal microscopy images of GL261 cells post 2‐hour Cu‐His NPs treatment, with nuclei stained blue by DAPI, lysosomes stained green with Lysotracker, and Cy5‐labeled Cu‐His NPs fluorescing red. B) Quantification of intracellular GSH and GSSG levels in GBM cells following a 24‐hour exposure to the agents. (*n* = 3; one‐way ANOVA, Tukey's multiple comparisons test, ns: not significant, **P < 0.01, ***P < 0.001). C) Measurement of intracellular ATP levels in GBM cells after 24 h of treatment. (*n* = 3; one‐way ANOVA, Tukey's multiple comparisons test, ns: not significant, **P < 0.01, ***P < 0.001). D) ROS staining fluorescence in GBM cells after a 4‐hour incubation with the agents, was visualized with DAPI‐stained nuclei in blue and DCFH‐DA‐stained ROS in green. E) Release of CRT and F) HMGB1 from GBM cells after 24 h of treatment with the agents. Data are presented as the mean ± S.D. (*n* = 3; one‐way ANOVA, Tukey's multiple comparisons test, ns: not significant, **P < 0.01, ***P < 0.001).

Previous experiments demonstrated that Cu‐His NPs emitted purplish‐red fluorescence upon interaction with GSH. After a 2‐hour incubation, confocal microscopy revealed intracellular red fluorescence, indicating that the Cu‐His NPs had been internalized and initiated their reaction with GSH (Figure S18A, Supporting Information). By 8 h, the red fluorescence within the cells had nearly vanished, suggesting the completion of the Fenton‐like reaction of Cu‐His NPs (Figure S17B, Supporting Information). To further visualize the intracellular drug release process of Cu‐His NPs, lysosomes were labeled with Lysotracker Red and co‐localized with Cy5‐labeled Cu‐His NPs, which exhibited blue fluorescence, and the nucleus. After 4 h of incubation, the fluorescence of Cu‐His NPs almost entirely overlapped with that of the lysosomes. The overlapping region displayed yellow fluorescence, indicating that the Cu‐His NPs were sequestered in lysosomes following endocytosis, and some had escaped into the cytoplasm (**Figure** **3**A; Figure S19, Supporting Information). These findings confirmed that Cu‐His NPs could undergo lysosomal escape post‐cell entry, releasing the drug into the cytoplasm and enhancing its intracellular efficacy. The mechanism of intracellular escape by Cu‐His NPs likely involved synergistic effects of histidine properties within the specific environment of tumor cells.^[^ ^34^ ^]^ Histidine's protonation in the lysosome's acidic milieu and its subsequent charge change may have facilitated its interaction with the lysosomal membrane and promoted escape. Additionally, histidine's transition from hydrophobic to hydrophilic properties, along with its proton sponge effect, facilitated the efficient uptake and swift drug release of Cu‐His NPs. These effects collectively promote the efficient uptake of Cu‐His NPs within cells, enhance the bioavailability of Cu^2+^, and enhance anti‐tumor effects.^[^ ^35^ ^]^

ROS in excess exerted a complex impact on tumor cells, disrupting redox balance and inducing damage or death. This triggered ICD, releasing DAMPs that activated antigen‐presenting cells, including dendritic cells and stimulated the immune system. GSH was critical for countering ROS‐induced oxidative stress; its levels inversely correlated with ROS, and reduced GSH could lead to ROS accumulation and cell death. Our study assessed the effects of different formulations on GSH and GSSG levels in GL261 cells. Both CB‐839 and Cu‐His NPs individually reduced GSH levels to 19.0 and 11.7 µm, respectively. However, the synergistic effect of CB‐839 and Cu‐His NPs more significantly blocked both the synthesis of new GSH and the depletion of pre‐existing GSH, with an effective concentration of just 8.2 µm (Figure 3B). Subsequently, ROS production in tumor cells post‐treatment was monitored using DCFH‐DA staining. The Combo group exhibited the highest ROS production, with a marked increase in fluorescence intensity (Figure 3D). In addition, DAMPs associated with ICD, such as surface calreticulin (CRT), high mobility group box 1 protein (HMGB1), and ATP, were released during cell death.^[^ ^36^ ^]^ ATP emitted “find‐me” signals, promoting the phagocytosis of apoptotic cells and triggering specific anti‐tumor immune responses.^[^ ^37^ ^]^ CRT served as an “eat‐me” signal, enhancing the phagocytosis of apoptotic cells by dendritic cells or their precursors, thereby providing antigens and promoting DC maturation and function.^[^ ^38^ ^]^ HMGB1 is bound to pattern recognition receptors on bone marrow cells, activating immune signaling pathways and further amplifying immune responses.^[^ ^39^ ^]^ The experimental results revealed that the Combo group significantly increased the expression of DAMP signaling molecules in tumor cells compared to the single drug treatment group (Figure 3C,E,F). The Combo therapy not only enhanced ICD by promoting the release of DAMPs from GBM cells but also amplified the immune response, suggesting a potential for long‐lasting anti‐tumor immunity and a promising approach for cancer therapy.

### 2.3 In Vivo Anti‐Tumor Studies of Combo Gel

**Figure 4:**
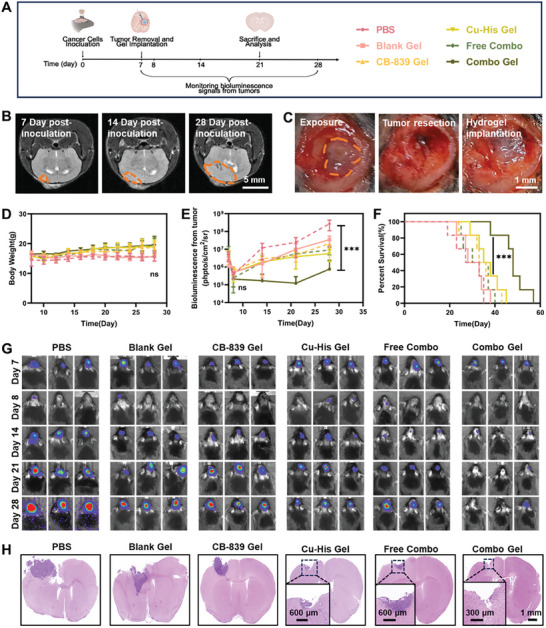
Anti‐tumor recurrence efficacy of different treatments in an orthotopic GBM model following resection. A) Schematic of the animal study timeline. B) T2‐weighted MRI images of orthotopic GBM mice with Luc‐GL261 at various time points. Experiments were repeated three times. The tumor mass is outlined in orange. C) Surgical debulking of mice with orthotopic Luc‐GL261 cells on day 7 post‐tumor implantation. The GBM mass is outlined in orange. D) Post‐treatment changes in mouse body weight, and E) tumor bioluminescence over time. Data are presented as mean ± SEM. (*n* = 6; one‐way ANOVA, TuKey's multiple comparisons test, data from the same point in time were statistically analyzed, ns: no significant, ***P < 0.001). F) mouse survival curves after different treatments. (Log‐rank test, ***P < 0.001) G) Temporal changes in bioluminescence signals from Luc‐GL261 tumors across treatment groups (partial data). Data are expressed as mean ± SEM (*n* = 6; one‐way ANOVA, Tukey's multiple comparisons test, ***P < 0.001). H) Two weeks following surgical resection and in situ drug treatment, brain slices from mice were obtained for histological analysis using H&E staining. Experiments were repeated three times.

The infiltrative growth of GBM often results in indistinct tumor boundaries, making complete surgical removal challenging and leading to frequent post‐surgical recurrence. A primary GBM model was successfully established using Luc‐GL261 cells (**Figure** **4**A,B; Figure S20, Supporting Information). Mice with similar tumor growth were selected and underwent resection on the seventh day after inoculation with Luc‐GL261 cells (Figure 4C). The mice were treated with either PBS, blank Fmoc‐YD/YYK hydrogel (Blank Gel), CB‐839 hydrogel (CB‐839 Gel), Cu‐His NPs hydrogel (Cu‐His Gel), free CB‐839 and Cu‐His NPs (Free Combo), or CB‐839 and Cu‐His NPs hydrogel (Combo Gel). Tumor recurrence was tracked by monitoring bioluminescent signals, and the mice's body weights and survival rates were recorded throughout the study. The PBS‐treated mice experienced rapid GBM recurrence and subsequent weight loss due to the absence of drug intervention. The Free Combo group initially lost weight, likely due to the rapid release of a high drug dose. In contrast, the CB‐839 Gel, Cu‐His Gel, and Combo Gel groups showed minimal weight changes, suggesting that drug‐loaded hydrogels effectively suppressed GBM growth without significant toxic side effects (Figure 4D). Post‐surgical mice with bioluminescence intensity between 10^6^ and 10^7^ were selected for surgery, and the success of the procedure was confirmed by re‐monitoring bioluminescence on day 8.^[^ ^40^ ^]^ Tumor bioluminescence was monitored every 7 days until the PBS group's mouse count dropped below three. The Combo Gel group exhibited the most pronounced inhibitory effect on GBM growth, with the lowest luciferase intensity across all monitoring points (Figure 4E,G). The Kaplan–Meier survival curves revealed that the median survival time for the Combo Gel‐treated group was significantly extended to 48 days, surpassing the 27 days observed in the PBS group, 28 days in the Blank Gel group, 33 days in the CB‐839 Gel group, 35 days in the Cu‐His Gel group, and 30 days in the Free Combo group (Figure 4F). Figure 4H displays H&E‐stained brain tissue sections from mice two weeks post‐surgical resection and in situ administration of the various treatments. In comparison to the control group, mice treated with Combo Gel exhibited the smallest tumor area, suggesting its superior efficacy in preventing tumor recurrence. In summary, the experimental results demonstrated that the Combo Gel treatment, which integrates glutamine metabolism regulation with CDT, provided outstanding therapeutic outcomes. It effectively suppressed the recurrence of GBM tumors, extended the median survival of mice, and induced systemic anti‐tumor immunity in vivo.

### 2.4 In Vivo Tumor Immune Effect Study of Combo Gel

**Figure 5:**
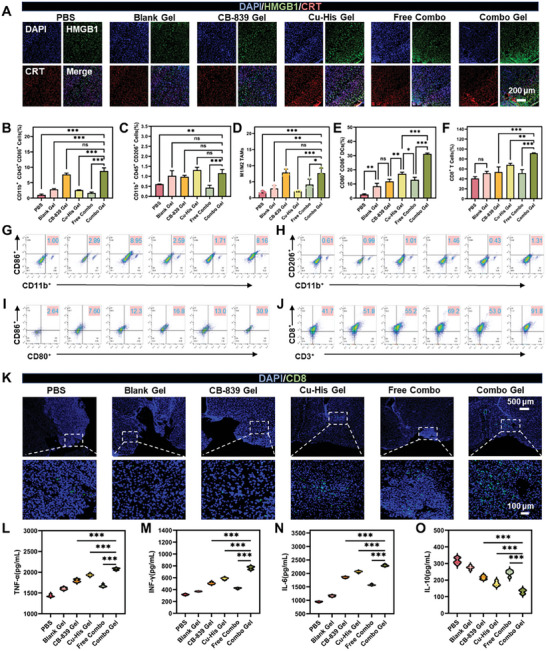
Activate anti‐tumor immunity post‐treatment with different agents. A) Immunofluorescence analysis of HMGB1 and CRT. B) Flow cytometry analysis of M1‐type TAMs, C) M2‐type TAMs, D) the M1/M2 TAM ratio, E) mature DCs, and F) CD8 T cells in mouse brain tissue two weeks post‐resection and in situ drug delivery (*n* = 3; one‐way ANOVA, Tukey's multiple comparisons test, ns: not significant, *P < 0.05, **P < 0.01, ***P < 0.001). Flow cytometry quantification of infiltrating G) M1‐type TAMs, H) M2‐type TAMs, I) mature DCs and J) CD8 T cells in brain tissue across groups. K) Immunofluorescence analysis of CD8 T cell infiltration in mouse tumor tissue. ELISA quantification of L) TNF‐α, M) IFN‐γ, N) IL‐6, and O) IL‐10 in mouse brain tissue homogenates for each group (*n *= 5; one‐way ANOVA, Tukey's multiple comparisons test, ns: not significant, *P < 0.05, **P < 0.01, ***P < 0.001).

The tumor‐killing process of ROS‐induced ICD is closely related to the production of related DAMPs, and HMGB1 and CRT are typical biomarkers associated with DAMPs^[^ ^41^ ^]^ **Figure** **5**A demonstrated that after two weeks of in situ administration of each group of gel biomaterials following surgical resection, the Combo Gel group exhibited the highest fluorescence signal intensity for CRT and HMGB1 in the brain. Notably, the expression of HMGB1 (green fluorescence) was significantly upregulated in the Combo Gel group, effectively promoting the maturation of DC cells in vivo. When comparing the fluorescence signals among the CB‐839 Gel, Cu‐His Gel, and Combo Gel groups, the CB‐839 Gel group showed lower DAMP expression than the other two groups, suggesting that the enhanced ICD effect was primarily due to the excessive ROS production by Cu‐His NPs within tumor cells.

Symbiotic cells contribute to the development and metastasis of GBM through metabolic interactions, including substrates and products. Metabolic competition for nutrients between tumor cells and immune cells can significantly influence antitumor immunotherapy.^[^ ^42^ ^]^ Since tumor cells needed to consume large amounts of glutamine for energy supply, their demand was much higher than that of normal cells and immune cells, a condition known as “glutamine addiction”. Consequently, the inhibition of GLS1 in tumor cells by the glutaminase inhibitor CB‐839 not only changed the metabolic pattern of GBM but also assisted in reversing the excessive glutamine consumption in the tumor environment. This provided nutrients for immune cells to more effectively carry out their tumor‐killing function. Cellular metabolism regulation not only inhibits tumor cell proliferation and survival but also impacts immune cell function and state. In this system, reprogramming metabolic processes breaks the communication network between the metabolic cycle and downstream immune microenvironment, and affects the activity and function of immune cells, discovering and eliminating residual tumor cells after surgery.

CB‐839 is a small molecule inhibitor of GLS1, which has been proven in vitro to block the significant consumption of glutamine by tumor cells. When the content of glutamine increases, it reduces the competition pressure between immune cells and tumor cells for glutamine, promotes T cell proliferation and cytokine production, and affects macrophage polarization. Initially, we assessed the polarization of TAM using flow cytometry analysis. The findings revealed a notable increase in the proportion of M1‐type TAM and a marginal rise in M2‐type TAM percentages in both the PBS and Combo Gel groups (Figure 5B–H). Further analysis indicated that the M1/M2 TAM ratio rose to 23.6 ± 1.0 and 23.1 ± 1.5 in the CB‐839 Gel and Combo Gel groups, respectively, which was 4.6 times greater than that observed in the PBS group (5.0 ± 0.4) (Figure 5D). The experimental outcomes suggested that CB‐839′s modulation of the glutamine metabolic pathway could induce the polarization of M2‐type TAM to M1‐type TAM, potentially reshaping the tumor immune microenvironment. The high infiltration of M2‐type TAMs in recurrent GBM is a well‐recognized characteristic, and our approach aims to counteract this by modulating macrophage polarization. However, the subtle changes in the number of M2 TAMs observed in our study warrant further investigation. It is hypothesized that the presence of copper‐based nanomaterials in Combo Gel may contribute to the increased infiltration of M2 macrophages. Similar phenomena have been observed in other emerging nanomaterials containing copper ions, where a shift toward the M2 macrophage phenotype has been noted.^[^ ^43^ ^]^ The elevated levels of ROS may play a pivotal role in increasing the number of M2 TAMs by modulating the Stat3 signaling pathway.^[^ ^44^ ^]^ Additionally, the complex tumor microenvironment and the presence of other immune cells and factors may counteract the effects of Combo Gel.^[^ ^45^ ^]^ There is also a possibility that other immune cells may compensate for the reduced M2 TAMs, maintaining an overall immunosuppressive environment.^[^ ^46^ ^]^ In summary, while Combo Gel shows a less pronounced effect on reducing the number of M2 TAMs, it does induce a shift toward a more immunoactive macrophage phenotype.

As antigen‐presenting cells, the activation of dendritic cells (DCs) is a pivotal step in initiating T‐cell responses and establishing anti‐tumor immunity. Encouragingly, the Combo Gel group exhibited a significantly higher percentage of activated DCs. In locally recurrent tumor tissues, the DC cell count in the Combo Gel group increased twelvefold compared to the PBS group (Figure 5E,I), indicating a reduction in the immunosuppressive microenvironment post‐treatment. CD8^+^ T cells, upon activation, differentiate into cytotoxic T lymphocytes that specifically target and eliminate tumor cells, forming the cornerstone of the adaptive immune system. The presence of CD8^+^ T cells is crucial for preventing tumor recurrence. To confirm whether Combo Gel effectively activated and recruited CD8^+^ T cells, we conducted flow cytometry and immunofluorescence analyses on tumor tissues. As anticipated, the Combo Gel‐treated tumors showed a marked increase in CD8^+^ T cell infiltration compared to the PBS group (Figure 5F,J). Interestingly, the Cu‐His Gel group also significantly attracted CD8^+^ T cells around the resection site (Figure 5K). However, the effect was notably diminished in the Free Combo group, which lacked the gel scaffold, suggesting that the sustained drug release in the Combo Gel group facilitated T‐cell infiltration, effectively reshaped the tumor immune microenvironment, and achieved long‐term local immune modulation. The molecular mechanisms by which immune cells such as CD8+ T cells are recruited to the periphery of tumors, a process that is highly complex, and the subsequent fate of these recruited immune cells, remain unclear.

We collected and analyzed mouse brain tissue homogenates for tumor necrosis factor‐α (TNF‐α), interferon‐γ (IFN‐γ), interleukin‐6 (IL‐6), and interleukin‐10 (IL‐10) using enzyme‐linked immunosorbent assay (ELISA) in each treatment group. The cytokine concentrations in the Combo Gel group were significantly different from those in other treatment groups. The Combo Gel group exhibited increased levels of pro‐inflammatory cytokines TNF‐α, IFN‐γ, and IL‐6 (Figure 5L,N), while the concentration of the anti‐inflammatory cytokine IL‐10 was reduced (Figure 5O). TNF‐α, primarily secreted by activated macrophages, can directly kill tumor cells. The Combo Gel group showed a marked increase in TNF‐α expression to 2076.9 ± 29.4 pg mL^−1^, which was 1.4 times higher than the PBS group. IFN‐γ, known to promote antigen processing and delivery, inhibit tumor cell growth, and enhance systemic anti‐tumor immunotherapy, was expressed at 765.3 ± 25.1 pg mL^−1^ in the Combo Gel group, a 2.4‐fold increase over the PBS group. IL‐6 expression in the Combo Gel group was 2293.0 ± 29.4 pg mL^−1^, which was 2.4 times and 2.0 times higher than that in the PBS group (943.7 ± 18.0 pg mL^−1^) and the Blank Gel group (1161.6 ± 27.1 pg mL^−1^), respectively, while IL‐10 expression was lower than in the PBS group. The above experiments indicate that Combo Gel not only increases the expression of pro‐inflammatory cytokines but also inhibits anti‐inflammatory cytokines, which help regulate the function of immune cells. In summary, immunotherapy based on glutamine metabolic regulation synergized with CDT to enhance T‐cell infiltration, promote TAM polarization, and elicit an immune response in mice.

## 3 Conclusion

The unique challenge of primary GBM lies in the presence of the BBB, which prevents many drugs from reaching the brain. Clinically, surgical resection is the most common standard treatment for GBM. By integrating our therapeutic strategy with surgical resection, we circumvent the issue of drugs struggling to cross the BBB to reach the brain. The direct application of the hydrogel at the resection site ensures a concentrated and sustained local delivery of the drug within the cranium, aiming to mitigate the recurrence of GBM. In conclusion, we have developed an immunotherapy strategy based on in situ peptide gel injection to cooperatively enhance CDT by metabolic reprogramming according to the specific requirements of postoperative treatment for GBM. The in situ injection hydrogel was first synthesized by controlling the assembly of short peptide molecules to fill the irregular cavity formed by surgical resection. We harnessed the self‐assembly coordination of Cu^2+^ with Fmoc‐HH to fabricate ROS‐generating Cu‐His NPs. Cu‐His NPs responded to the tumor‐specific microenvironment by depleting intracellular GSH and H_2_O_2_, generating ROS and disrupting the redox balance of tumor cells. By inhibiting glutaminase activity through CB‐839, GSH production was suppressed, thereby synergistically amplifying oxidative stress. Our experimental findings suggest that the introduction of Cu‐His NPs amplifies the ICD effect, while CB‐839 polarizes macrophages by modulating the glutamine metabolism pathway. These two drugs work in synergy to regulate the downstream immune microenvironment, enhance T cell infiltration and M2 macrophage activation in mice, initiate immune responses, and result in long‐term regulation of the local immune microenvironment. These results demonstrate the high efficiency of combined gels as a postoperative GBM treatment system and its great potential for clinical application.

The authors have cited additional references within the Supporting Information.^[^ ^47^, ^48^ ^]^

## Conflict of Interest

The authors declare no conflict of interest.

## Supporting information



Supporting Information

## Data Availability

The data that support the findings of this study are available from the corresponding author upon reasonable request.
