# Supplementary material for: Immunostimulatory Hydrogel with Synergistic Blockage of Glutamine Metabolism and Chemodynamic Therapy for Postoperative Management of Glioblastoma
Source: Adv Sci (Weinh). 2025 Feb 20;12(15):2412507. doi: 10.1002/advs.202412507 (PMC12005773; doi:10.1002/advs.202412507)
Supplement: Supplementary file 1 — Supporting Information [file ADVS-12-2412507-s001.docx]

**Supporting Information**

Immunostimulatory hydrogel with synergistic blockage of glutamine metabolism and chemodynamic therapy for postoperative management of glioblastoma

Yiran Guo,^[a, b]^ Tianhe Jiang,^[a]^ Sen Liang, ^[a]^ Anhe Wang, ^[a, d]^ Jieling Li, ^[a, d]^ Yi Jia,^[c, d]^ Qi Li,*^[a, d]^, Jian Yin,*^[b]^ Shuo Bai,*^[a, d]^ and Junbai Li*^[c, d]^

[a] Y. Guo, T. Jiang, S. Liang, A. Wang, J. LI, Q. Li, S. Bai
State Key Laboratory of Biopharmaceutical Preparation and Delivery, Institute of Process Engineering, Chinese Academy of Sciences, Beijing 100190, China

[b] Y. Guo, J. Yin
Key Laboratory of Carbohydrate Chemistry and Biotechnology, Ministry of Education, School of Biotechnology
Jiangnan University
Wuxi, Jiangsu 214122 (China)

[c] Y, Jia, J. Li
Beijing National Laboratory for Molecular Sciences (BNLMS), CAS Key Lab of Colloid, Interface and Chemical Thermodynamics
Institute of Chemistry, Chinese Academy of Sciences
Beijing, 100190 (China)

[d] A. Wang, J. LI, Y, Jia, Q. Li, S. Bai, J. Li

University of Chinese Academy of Sciences

Beijing, 100049 (China)

E-mail: liqi@ipe.ac.cn; jianyin@jiangnan.edu.cn; baishuo@ipe.ac.cn; jbli@iccas.ac.cn.

**Materials and methods**

***Materials***

All amino acids were purchased from GL Biochem (Shanghai) Ltd. CB-839 (cat. no. C174237) was obtained from Shanghai Aladdin Biochemical Technology Co., Ltd. Cell Counting Kit-8, 2-(4-amidinophenyl)-6-indolecarbamidine dihydrochloride (DAPI), GSH and GSSG Assay Kit, Calcein acetoxymethyl ester and propidium iodide (Calcein AM/PI), 2′,7′-dichlorofluorescin diacetate (DCFH-DA), Lyso-Tracker Red, ATP Assay Kit, were purchased from Beyotime Institute of Biotechnology (Shanghai). FITC anti-mouse CD86 antibody (cat. no. 105005) was obtained from BioLegend. APC anti-mouse CD8a antibody (cat. no. E-AB-F1104E), APC anti-mouse CD45 antibody (cat. no. E-AB-F1136E), PE anti-mouse CD11c antibody (cat. no. E-AB-F0991D), FITC anti-mouse CD206 antibody (cat. no. E-AB-F1135C), FITC anti-mouse CD3 antibody (cat. no. E-AB-F1013C), APC anti-mouse CD86 antibody (cat. no. E-AB-F0994E), FITC anti-mouse CD80 antibody (cat. no. E-AB-F0992C), PE anti-mouse/human CD11b antibody (cat. no. E-AB-F1018D), were purchased from Elabscience. Calreticulin monoclonal antibody (cat. no. YM0090) and HMGB1 (PTR2339) mouse mAb (cat. no. YM4697) were purchased from Immunoway. Anti-CD8 alpha antibody (cat. no. AB4055) was purchased from Abcam. All other reagents and solvents were purchased from Sigma-Aldrich.

***Cell Lines, and Animals***

Luc-GL261 cell line and RAW 264.7 macrophage cell line were kindly gifted from Prof. Ruitian Liu (Institute of Process Engineering, Chinese Academy of Sciences, Beijing, China). The Luc-GL261 cells were cultured in DMEM (Gibco, Invitrogen) containing 10% fetal bovine serum (Gibco), with 1% L-Glutamine (Solarbio) and 1%HEPES (Solarbio). The RAW264.7 macrophages were cultured in RPMI Medium 1640 (Gibco) containing 10% fetal bovine serum (Gibco). Six-to-eight-week-old male C57BL/6J mice were purchased from SPF (Beijing) Biotechnology Co., Ltd. All the animal experiments in this work were conducted in accordance with protocols approved by the Animal Care and Use Committee of the Institute of Process Engineering, Chinese Academy of Sciences, and in compliance with the Chinese law on experimental animals (approval ID: IPEAECA2023035).

***Cu-His NPs Synthesis.***

6 mg/mL Fmoc-protected-histidine-histidine (Fmoc-HH) was dissolved in deionized water, and 1 M NaOH was added to the solution until pH = 9. CuCl_2_ aqueous solution (6 mg/mL) was added to the mixture and stirred overnight to form Cu-His NPs. After the reaction, the precipitate was collected by centrifugation (15,000 rpm, 15 min) and washed with water and alcohol. After the reaction, the precipitate was collected by centrifugation (15000 rpm, 15 min) and washed with water and alcohol. The prepared Cu-His NPs were then lyophilized and stored at -20 °C for subsequent experiments.

***Hydrogel Preparation.***

Fmoc-protected Tyrosine -Tyrosine -Lysine (Fmoc-YYK) were dispersed in Milli-Q water to prepare solutions at different concentrations. Fmoc-Tyrosine-Aspartic acid (Fmoc-YD) was first co-solubilized by a trace volume of DMSO and then added to Milli-Q water. Samples were prepared under vortexing and sonication conditions. After the self-assembly process, Fmoc-YD/YYK hydrogels were obtained, and Fmoc-YD was mixed with Fmoc-YYK in a ratio of 1:1.

***In vitro ROS generation.***

A 1 mM solution of GSH was mixed with a 1 mM solution of Cu-His NPs, to which a solution of 10 μg mL^-1^ Methylene blue (MB) and 10 mM H_2_O_2_ was added as an experimental group and other controls were set up, and the ·OH-induced degradation of MB was measured by the change in UV absorbance after 30 min.

***Complete release of Cu-His NPs from hydrogel.***

1 mL of hydrogel loaded with Cu-His nanoparticles was taken and soaked in 10 mL of ddH2O, incubated in a 37°C incubator for 12 hours, and the supernatant from the soaked hydrogel was collected. The supernatant was centrifuged at 12,000 rpm, and the pellet was subjected to SEM to observe any changes in the morphology of the nanoparticles.

***Hydrogel Degradation Study.***

1 mL of 5 mM YD/YYK pre-hydrogel solution configured in 100 nM sodium bicarbonate buffer (pH = 8.3) was mixed with 1.2 mg/mL Cy5-SE and incubated on ice overnight. Cy5-modified hydrogels were prepared to obtain Cy5. 100 μL of 5 mM hydrogel was injected subcutaneously into C57BL/6J mice. The remaining hydrogel in each mouse was photographed and recorded every 3 days using the IVIS, and the degree of degradation of the hydrogel was determined by detecting the fluorescence intensity of the hydrogel.

***Release of Drugs In Vitro.***

1 ml of pre-gel solution containing CB-839 and the pre-gel solution containing Cu-His NPs were injected into the bottom of the tube and sonicated to promote complete sol-gel transformation. 9 mL of PBS was slowly added on top of the gels. the experiments were carried out at 37 °C in a shaking bath at 60 rpm. At the time points of 0, 3, 6, 12, 24, 48, 60, 120, 168, 240, 312, and 432h, three samples were randomly selected for the determination of CB-839 and Cu-His NPs by HPLC with gradient elution using a UV detector at 240 nm and 224 nm, respectively.

***Immune Crosstalk Between Tumor Cells and Macrophages.***

The RAW 264.7 macrophage cell line was inoculated into large dishes at a density of 6 × 10^5/well and induced with IL-4, 30 ng/mL, for 48 h. LUC-GL261 tumor cells, were inoculated into 6-well plates at 6 × 10^5/well. After 12 h of adherence, the cells were co-cultured with 10 μg/mL CB-839 medium. The supernatant of LUC-GL261 tumor cells was aspirated and centrifuged at 1000 × g for 5 min to remove cell debris. RAW 264.7 macrophages were cultured with tumor cell supernatant for 24 h. The supernatant was aspirated and assayed by ELISA.

***Cellular Uptake of Cu-His NPs.***

The GL261 cells were seeded in 35mm confocal culture plates at a number of 1 × 105 per, cultured for 12 h, and then incubated with Cy5-labeled Cu-His NPs that were diluted with DMEM at concentrations ranging from 50 μg/mL to 200 μg/mL for 4 h at 37 °C, respectively. After that, the cells were washed three times with PBS. Cells were stained for lysosomes by 0.1 μL/mL lysotracker. The cells were washed three times with PBS and fixed with 4% formaldehyde for 15 min. Then, nuclei were then stained with 1 μg/mL DAPI. Finally, evaluate the endocytosis of Cu-His NPs by confocal laser scanning microscope (CLSM).

***Intracellular ROS Staining.***

After coculturing with different groups of drugs for 12 h, First, the cells were washed with PBS twice. Then, the cells were incubated with 2′,7′-dichlorodihydrofluorescein diacetate (DCFH-DA) (10 μM) for 1 h and washed three times with PBS and fixed with 4% formaldehyde for 15 min. Then, nuclei were then stained with 1 μg/mL DAPI. Finally, the ROS signal of the cells was observed by CLSM.

***Creation of the Orthotopic GBM Model.***

Mice were anesthetized, then immobilized on a stereotaxic apparatus, and a midline incision was made in the skin above the cranium. A burr hole was drilled at the right cranial hemisphere, 2.0 mm lateral to bregma. Then, 3 × 10^5^ Luc-GL261 cells were implanted at a depth of 2.5 mm from the dura. Suturing the skin after injection. The mice were maintained under the standard housing conditions, and the orthotopic GBM was monitored using bioluminescence imaging performed in the IVIS imaging system by injecting D-luciferin potassium salt (15 mg/mL) intraperitoneally in advance. On day 7 after inoculation, brain tumor-bearing mice were randomly assigned to five groups (n = 6).

***GBM Tumor Resection Model and Treatment.***

The intracranial GBM model was successfully established on day 8, and the mice were anesthetized and immobilized on a stereotaxic apparatus. Exposure of the previous burr hole, based on the location of the burr hole, a 3×3 mm window was opened in the skull with a cranial drill. Under a dissecting surgical microscope, as much of the visible tumor tissue as possible was removed. Then, 10 μL of formulation solution was injected into the resection cavity using a syringe, and the wound was closed with tissue glue after the injection. Randomly grouped mice were treated with PBS, blank gel, CB-839 gel, Cu-His gel, free combo, and combo gel. The administered doses were 50 μg CB-839 and 50 μg Cu-His per mouse.

***Safety studies of hydrogels in healthy mice.***

Healthy male C57BL / 6J mice at 6-8 weeks of age were anaesthetized, a burr hole was punched as described above, and then 10 μL of combo gel was injected intracranially into the mice using a syringe. The mice were executed at predetermined time points, and the brains and other major organs were harvested and fixed. slides were imaged and photographed using an optical microscope after H&E staining.

***Flow Cytometry Assay***

Flow cytometry analysis was used to assess immune cells in tumors. Briefly, mice were executed for two weeks after gel injection, and brain tumors were collected from the mice. The tissue was filtered through a 70 μm single-cell filter. Cells were collected by centrifugation with the addition of 10% erythrocyte lysate, then washed with PBS and stained with fluorescently labeled antibodies. After staining, cells were washed with PBS, fixed with 4% paraformaldehyde, detected by flow cytometry (Beckman, coulter), and analyzed by FlowJo Software v10.

***ELISA***

For secretory samples, the cell culture supernatant is selected and centrifuged at 1000×g for 20 minutes, and then the supernatant is extracted. For intracellular samples, cell lysates were selected. Cell lysate is used for intracellular samples as follows: trypsin digestion of adherent cells, centrifugation of the cells, washing with cold PBS three times, and physical lysis of the cells (ultrasonic crushing or repeated freezing and thawing). The specimen was centrifuged at 1500×g for 10 min at 4 ℃, and the supernatant was collected and set aside. The ELISA method was as follows: The plate was set up with standard wells and sample wells, 100 uL of different concentrations of standards were added to each standard well, 100 uL of samples to be tested were added to the sample wells, 100 uL of universal diluent was added to the blank wells, and the plate was covered with a membrane and incubated at 37 °C for 1 hour. Discard the liquid, without washing, directly add 100 uL of biotinylated antibody working solution to each well, cover the plate membrane, and incubate at 37 °C for 1 hour, then discard the liquid. Add 100 uL of enzyme conjugate working solution to each well, cover the plate membrane, and incubate at 37 °C for 30 minutes. Discard the liquid, add 90 uL of substrate (TMB) to each well, cover the plate membrane, and incubate at 37°C for 15min, avoiding light. 50 uL of termination solution was added, and the OD value of each well was measured at 450 nm within 15 min. ELISA kits were used to detect CRT (J&L Biological, #JL42550), HMGB1(J&L Biological, #JL13702), IL6 (J&L Biological, #JL20268), IL10 (J&L, #JL20242), TNF-α (J&L, #JL10484), and INF-γ (J&L, #JL10967).

***Statistical Analysis***

All results were presented as means ± SD. The two-tailed unpaired t-test was used to determine the statistical significance between two treatment groups and ANOVA was used for multiple comparisons. Survival was plotted using a Kaplan-Meier curve and assessed by a log-rank (Mantel-Cox) test. Statistical analysis was performed using GraphPad Prism software 5. *P ≤ 0.05, **P ≤ 0.01, ***P ≤ 0.001.


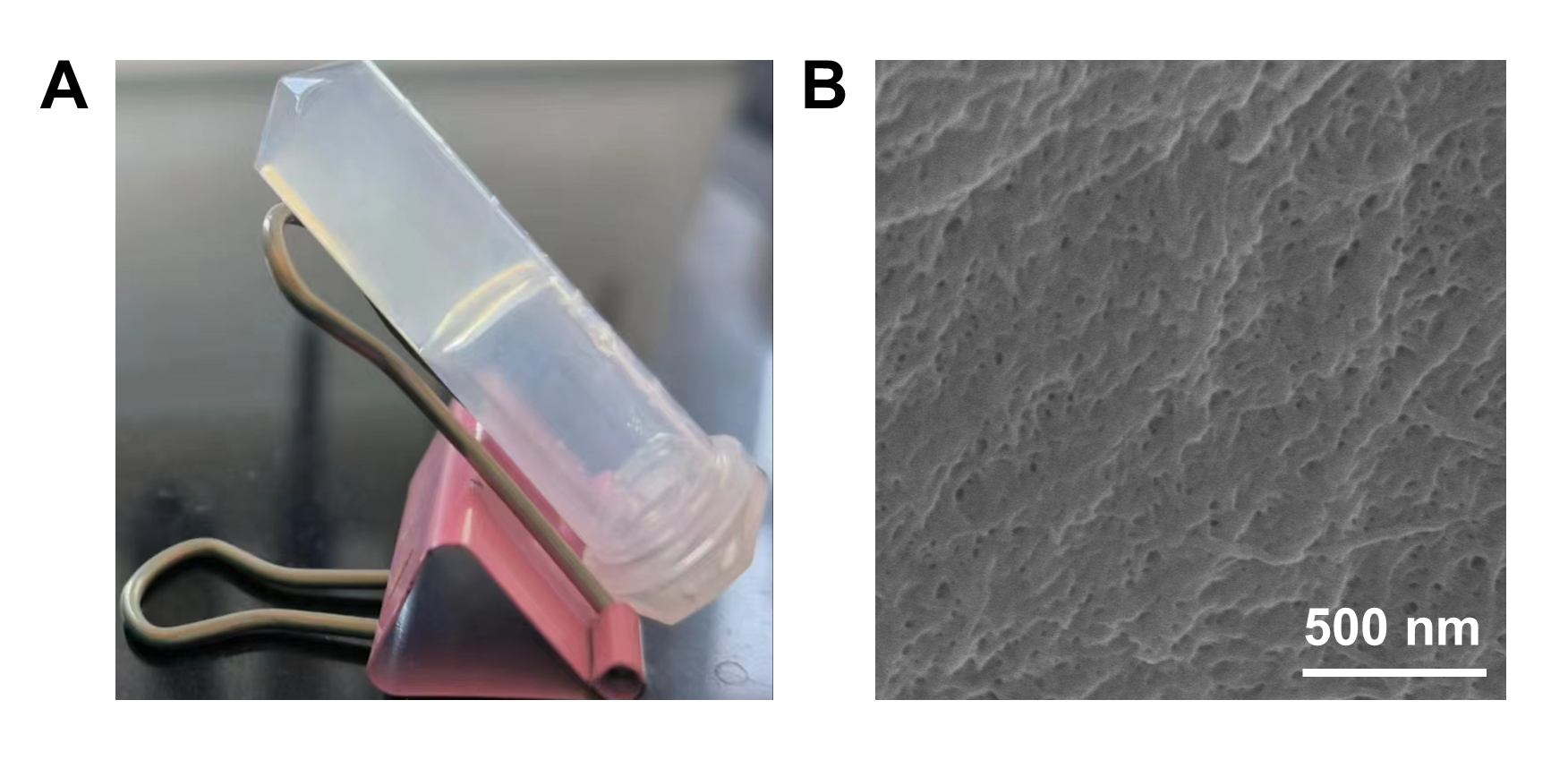


**Figure S1.** (A) Digital pictures of Fmoc-YD/YYK hydrogel. (B) SEM image of Fmoc-YD/YYK hydrogel.


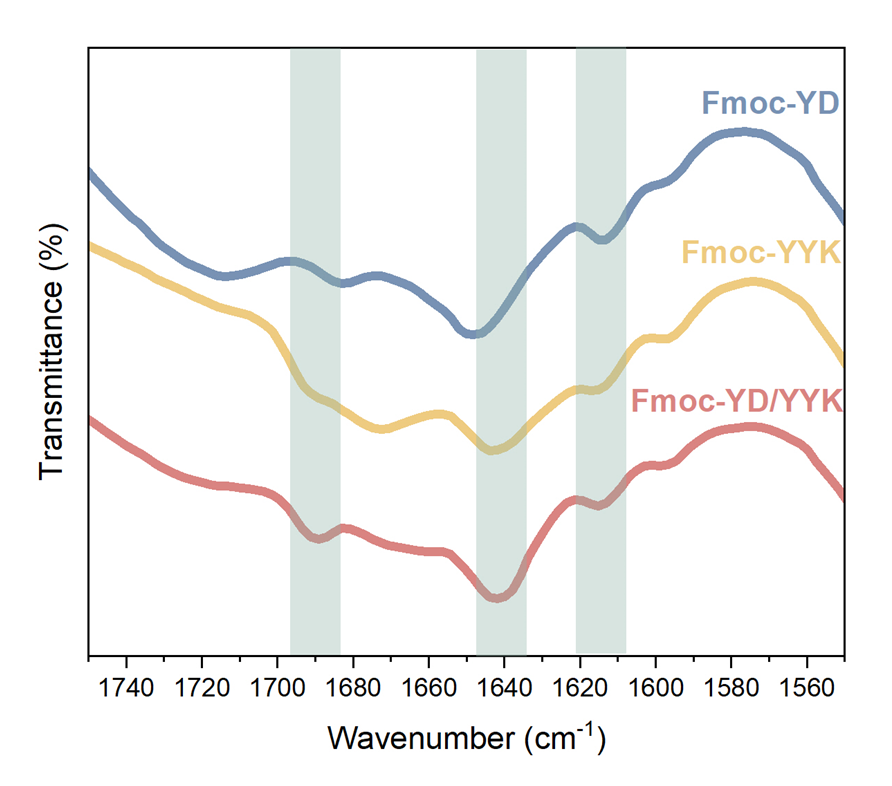


**Figure S2.** FT-IR spectra of Fmoc-YD/YYK hydrogel and its ingredients.


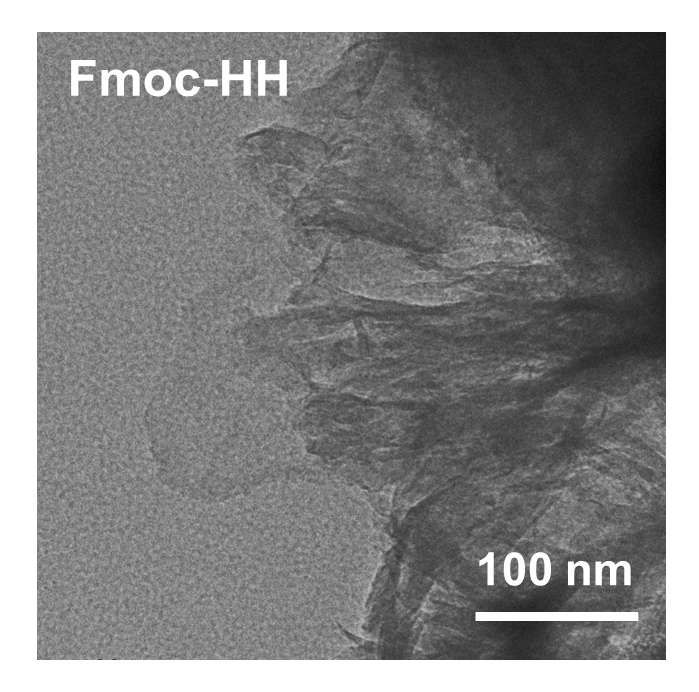


**Figure S3.** HRTEM images of Fmoc-HH.


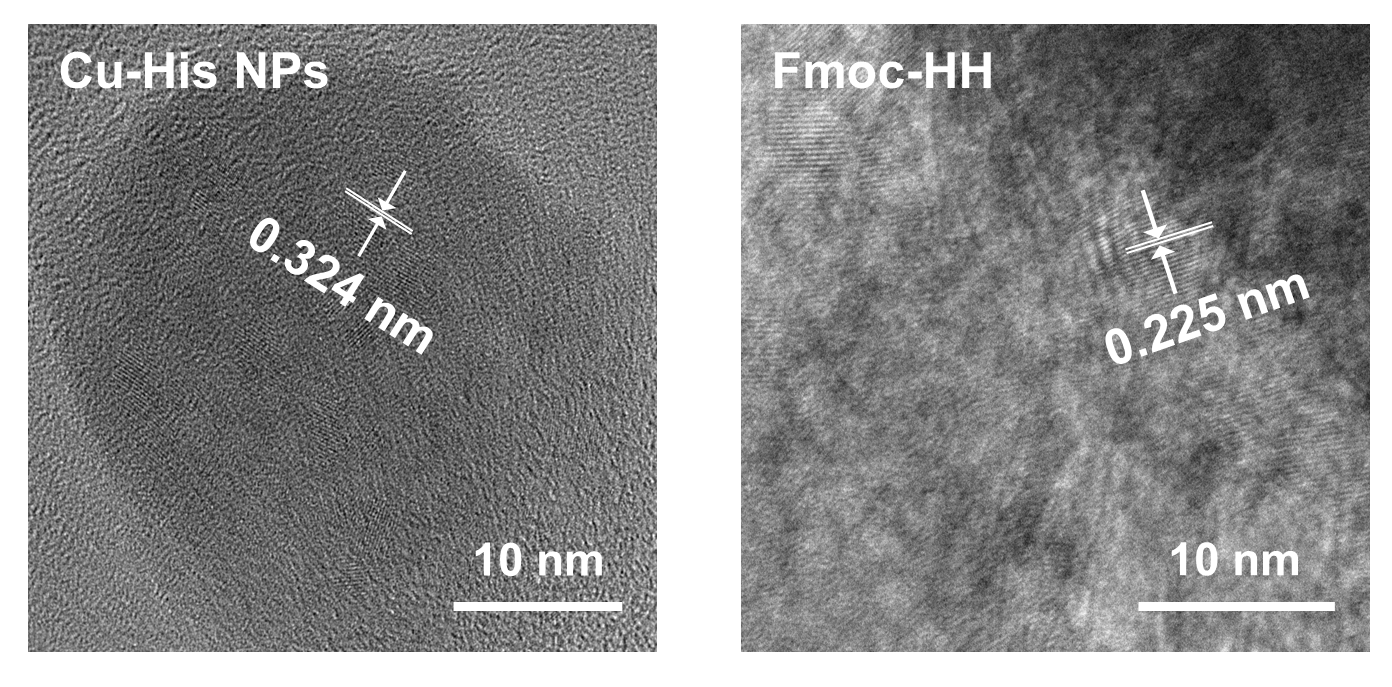


**Figure S4.** HRTEM images of Cu-His NPs and Fmoc-HH.


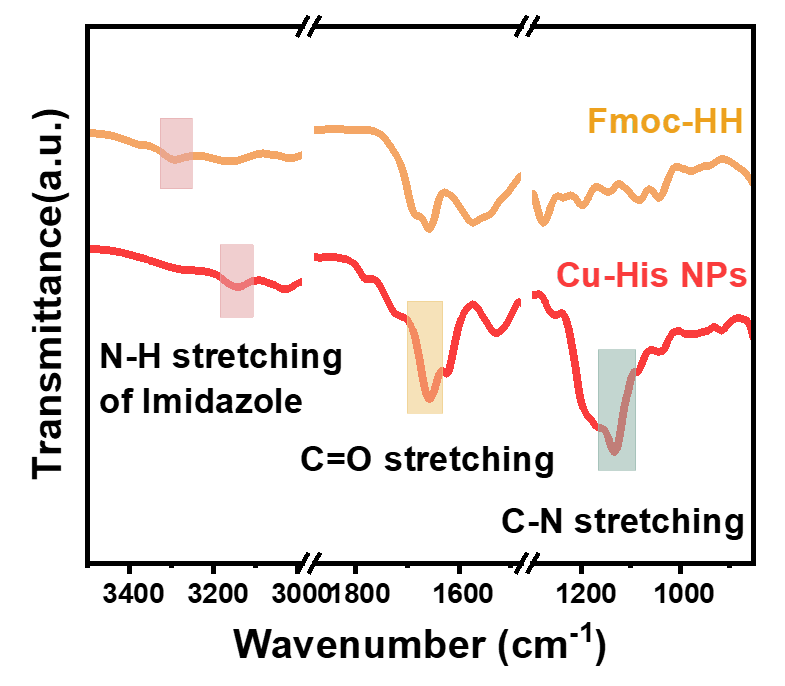


**Figure S5.** FT-IR spectra of Cu-His NPs and Fmoc-His-His (Fmoc-HH). The FT-IR spectra were vertically moved.


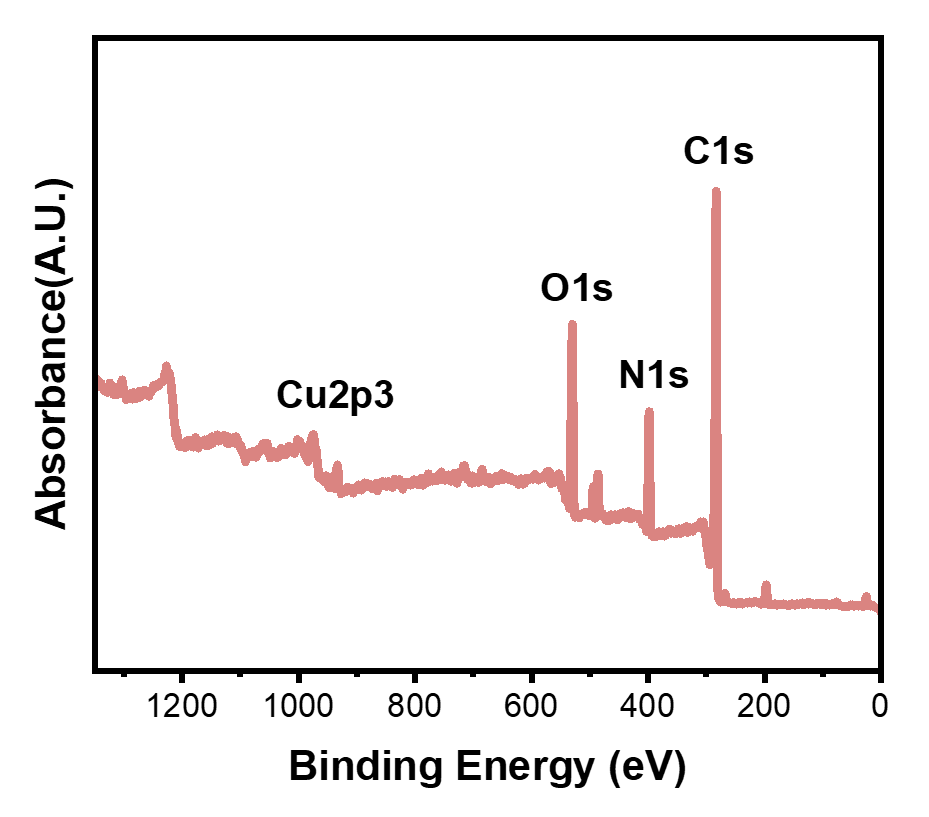


**Figure S6.** The XPS spectra of Cu-His NPs.


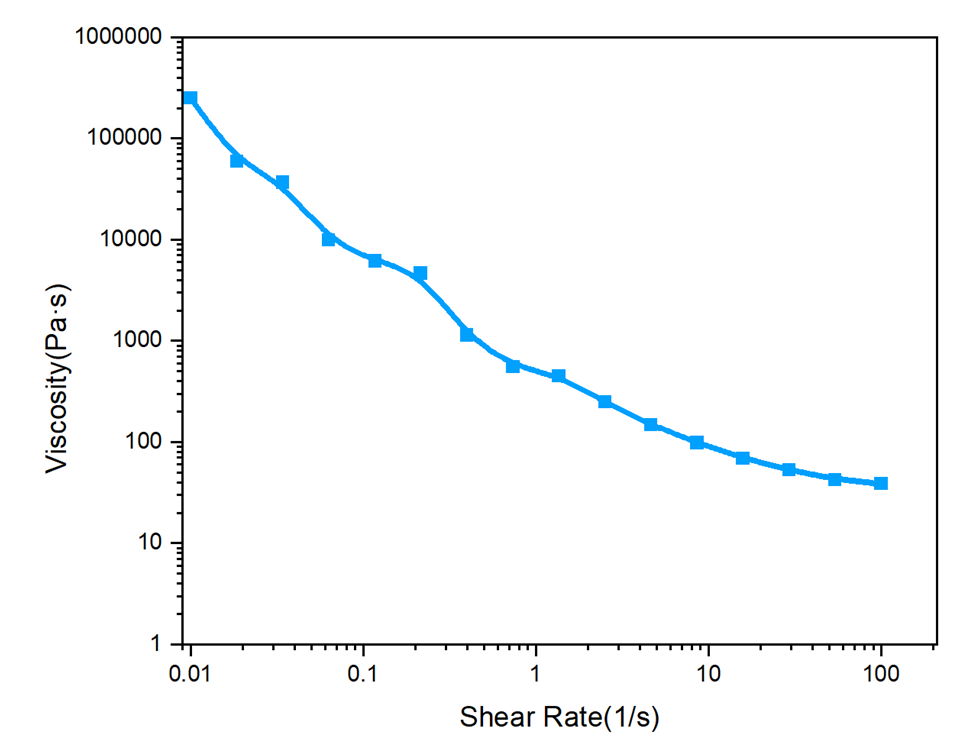


**Figure S7.** The viscosity of the 5 mM Combo Gel at a shear rate of 0.1 to 100 s^−1^.


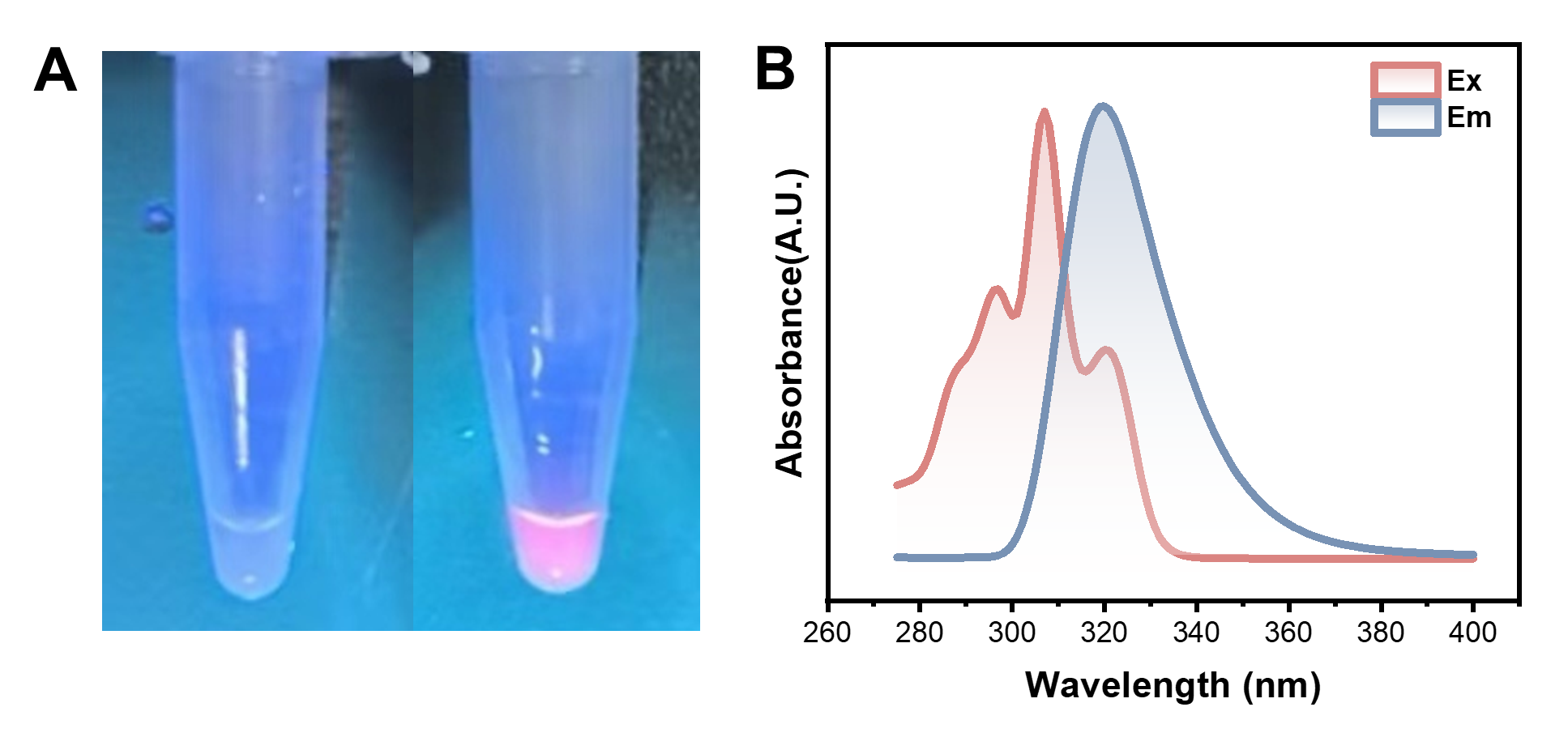


**Figure S8.** (A) Digital pictures of Cu-His NPs without (left) and with (right) GSH under a UV light. (B) Emission spectra and excitation spectra of Cu-His NPs mixed with GSH at a 1:1 mass ratio.


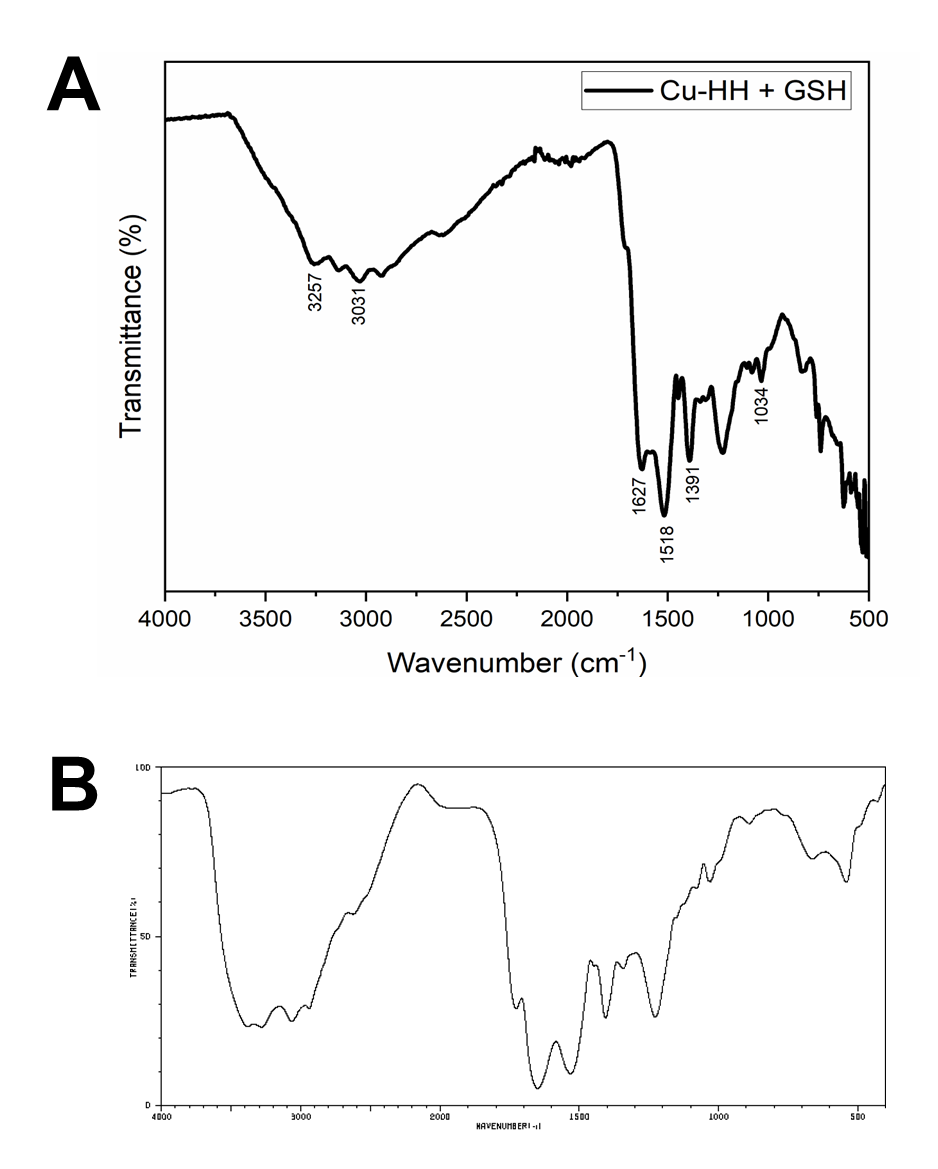


**Figure S9.** FT-IR spectra of products from the reaction of Cu-His NPs with GSH. ^[41]^

**
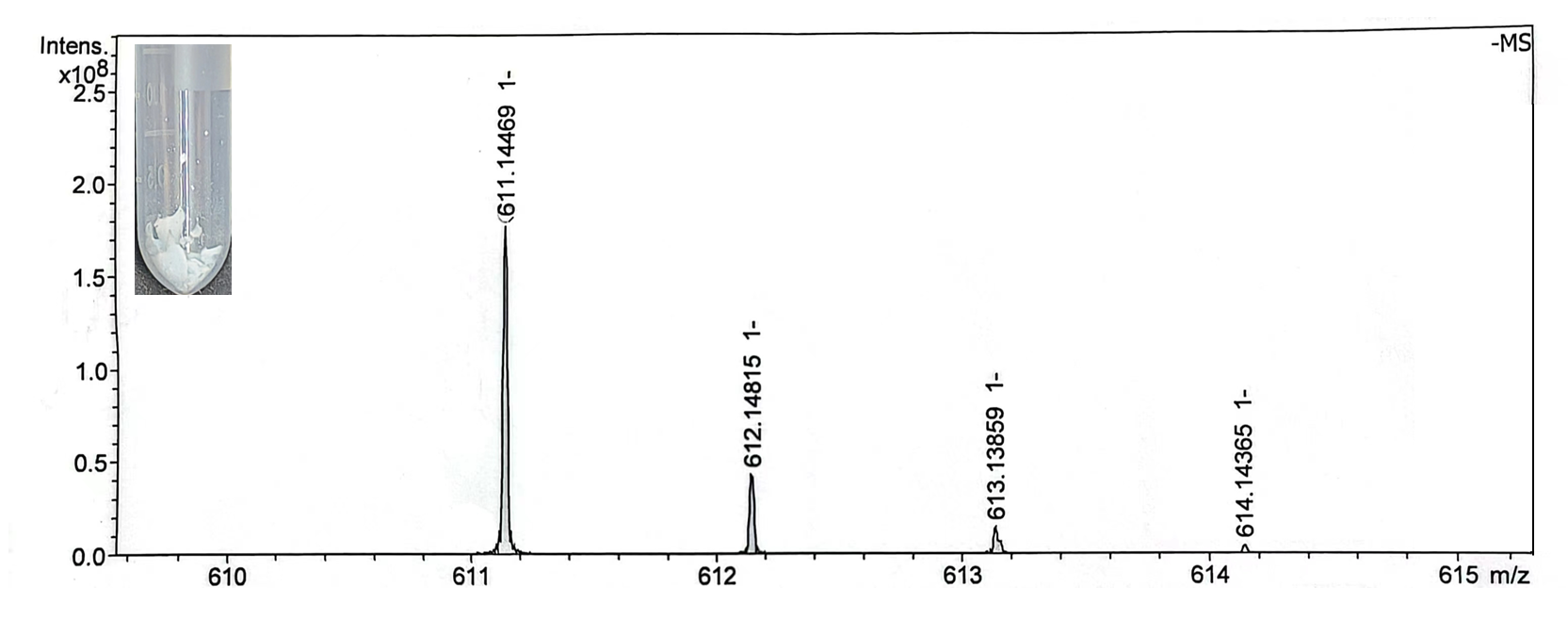
**

**Figure S10.** HR-MS of the product in D₂O. Digital picture of the white product, which was lyophilized from the reaction between Cu-His NPs and GSH.


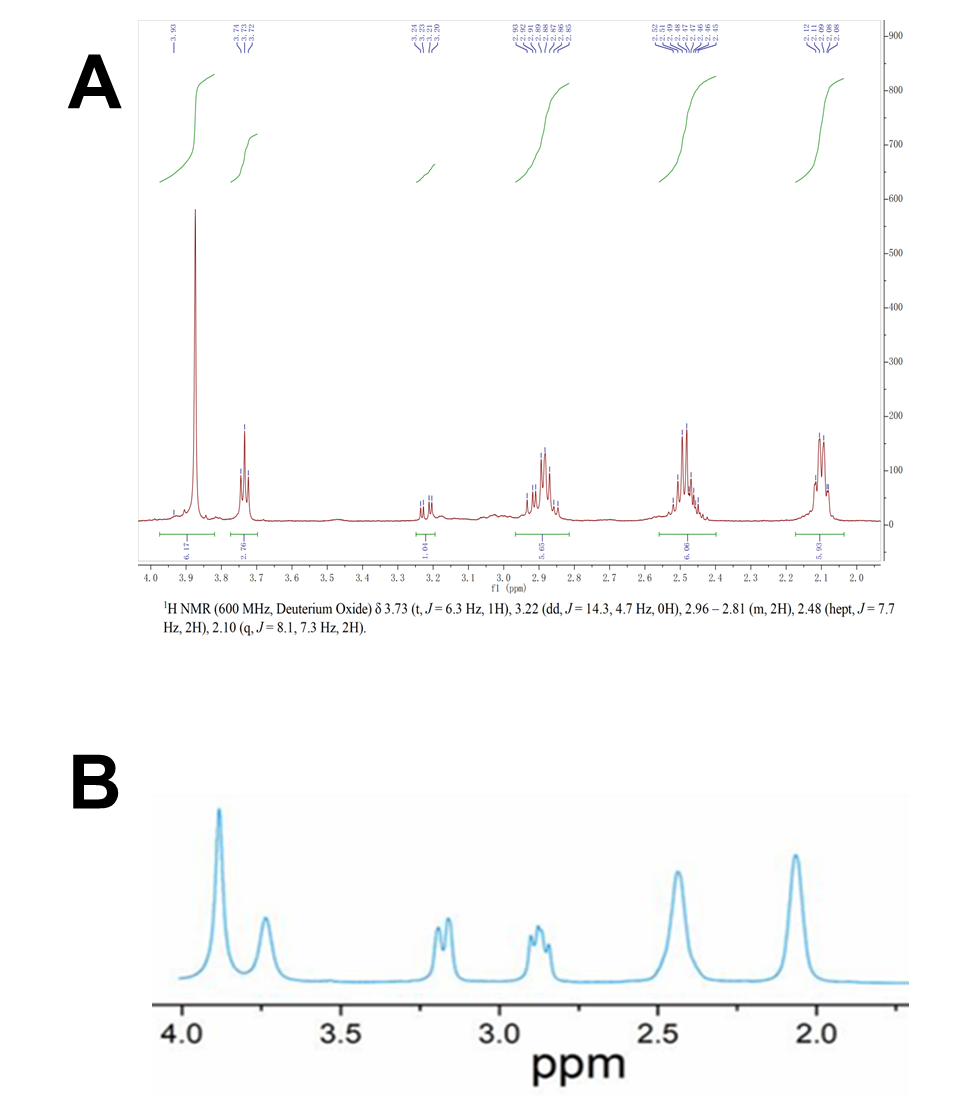


**Figure S11.** ^1^H-NMR of the products from the reaction of Cu-His NPs with GSH in D₂O. ^[42]^


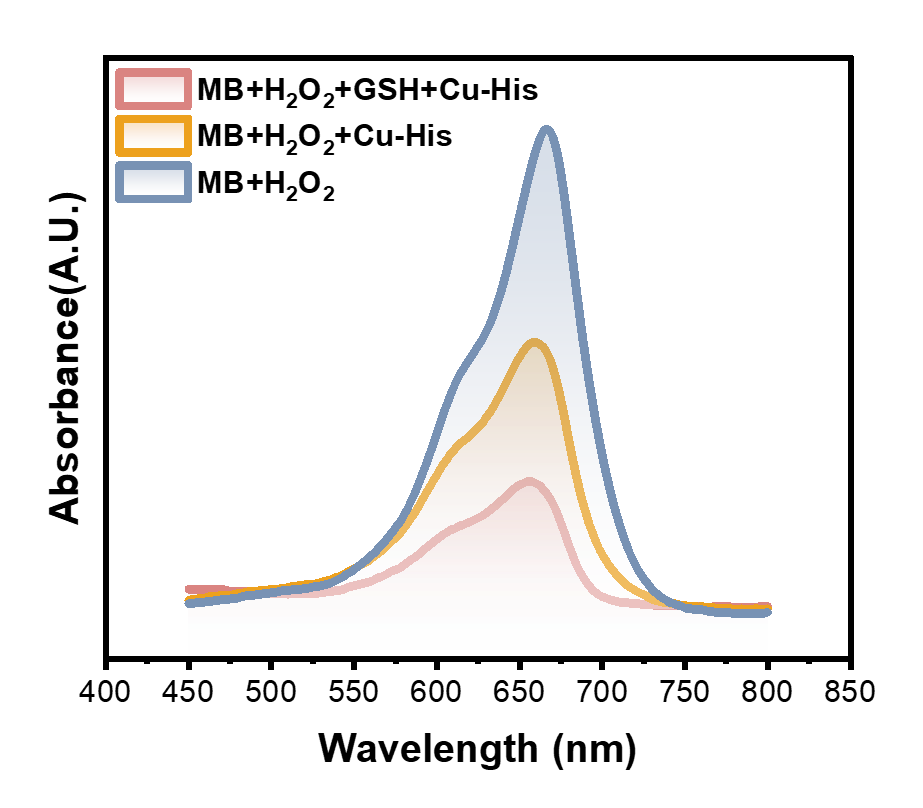


**Figure S12.** MB degradation shows the difference in the rate of Fenton-like reaction between Cu(II) and H_2_O_2_ in the presence or absence of GSH. The reaction time was 4 h.


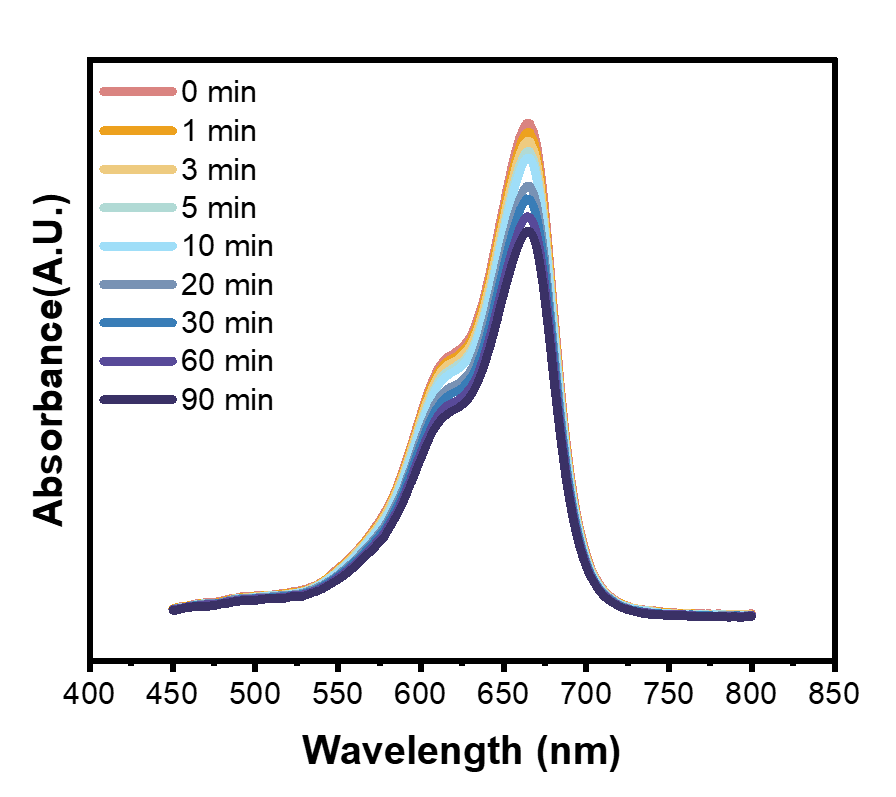


**Figure S13.** The MB degradation under the Cu-His NPs added H_2_O_2_ and GSH.

**
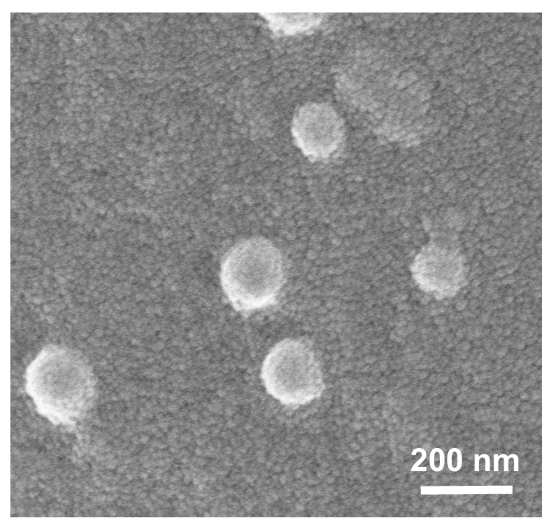
**

**Figure S14.** SEM image of the morphology of Cu-His NPs after release from the hydrogel.


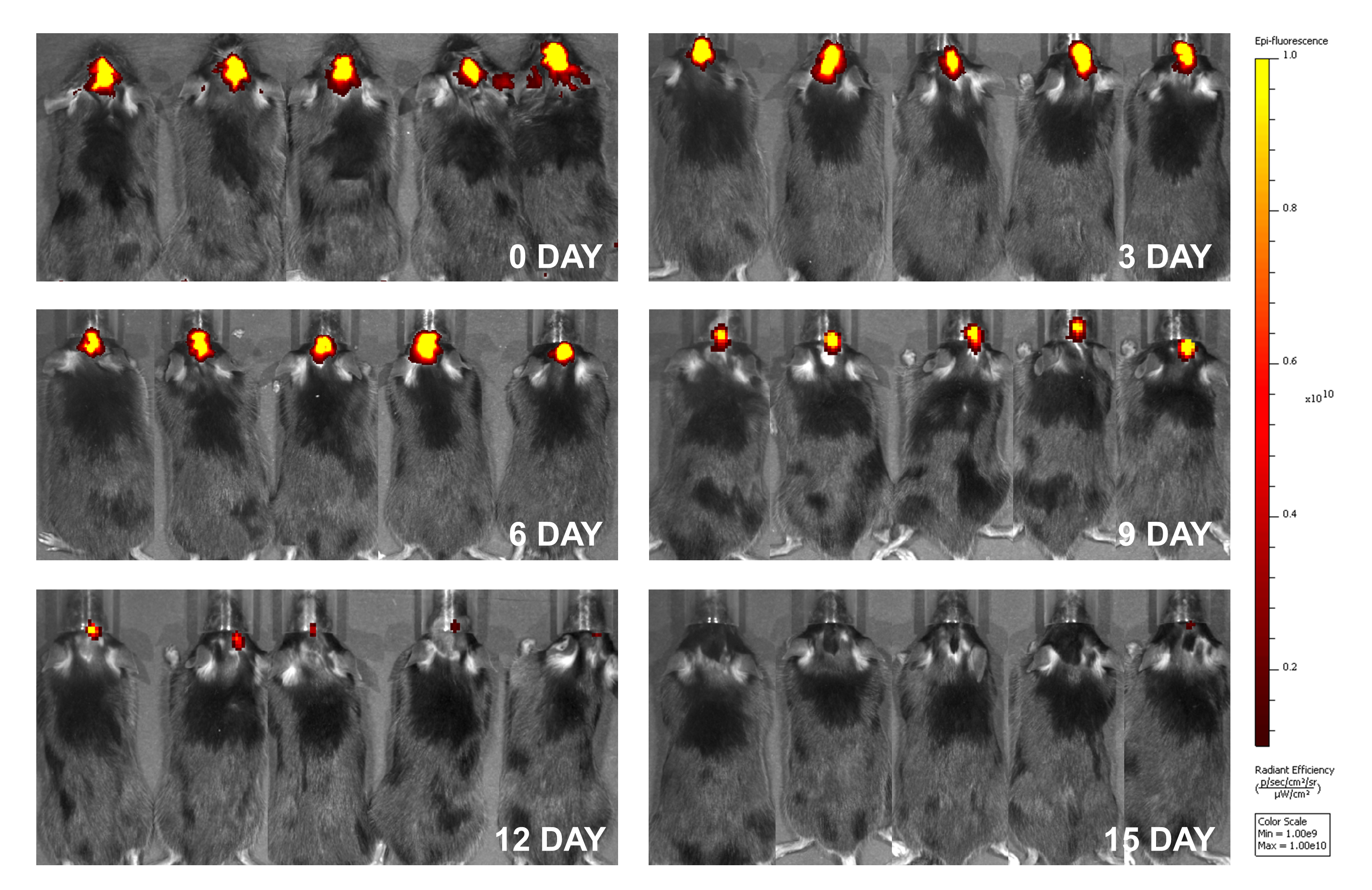


**Figure S15.** The IVIS images show retention of the hydrogel at the indicated time points after intracranial injection of the hydrogel, which was labeled with Cy5.


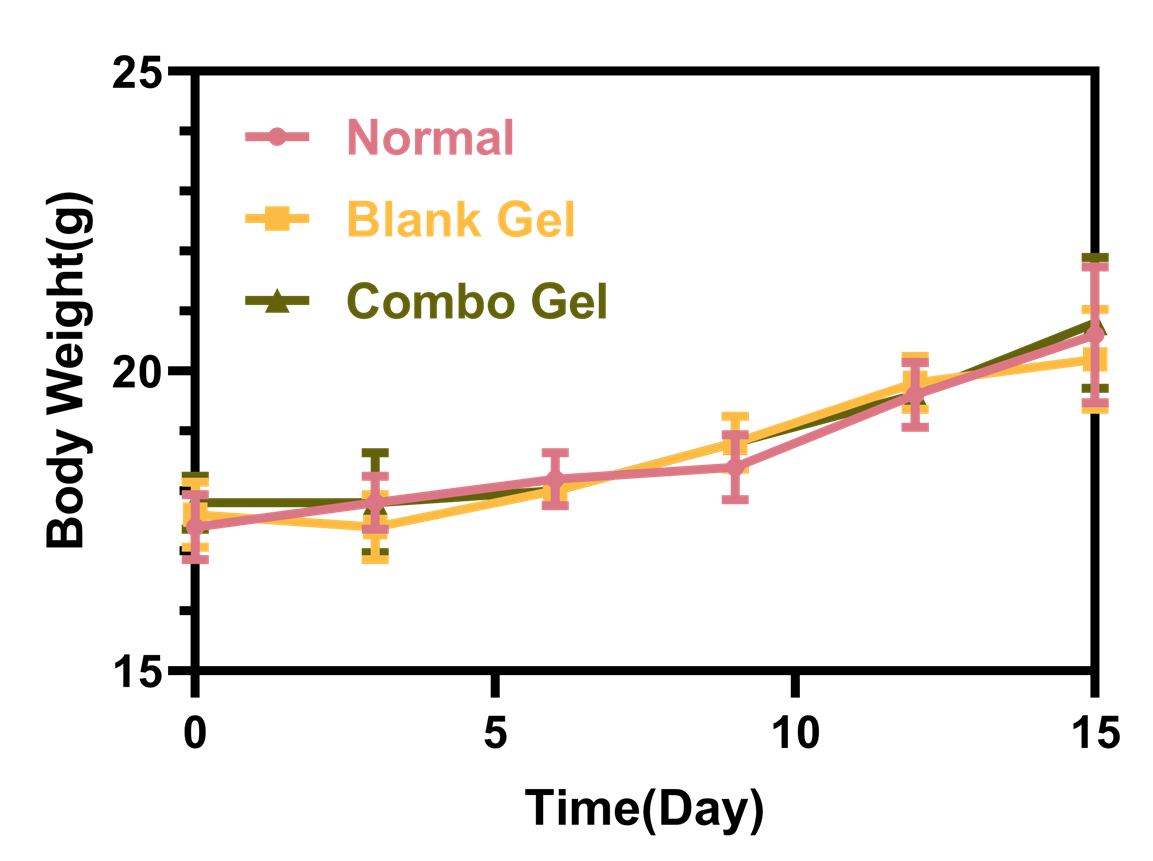


**Figure S16.** Body weight curves corresponding to indicate different groups. Data were presented as mean ± SEM (n = 5). (one-way ANOVA, TuKey’s multiple comparison test, the data at the same time point were statistically analyzed)


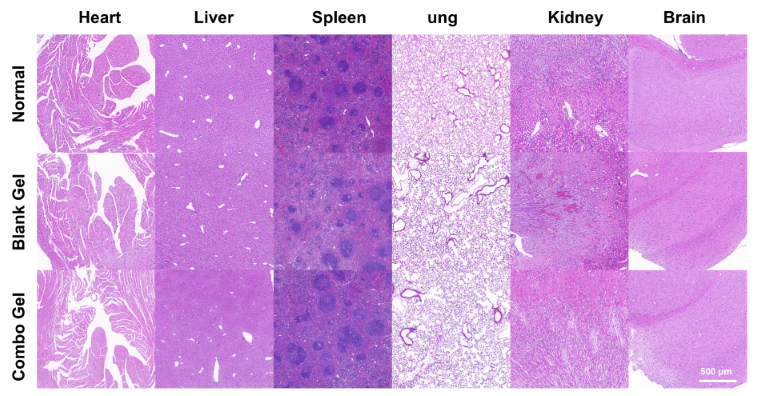


**Figure S17.** H&E staining images of major organ sections excised from mice treated with different formulations.

**
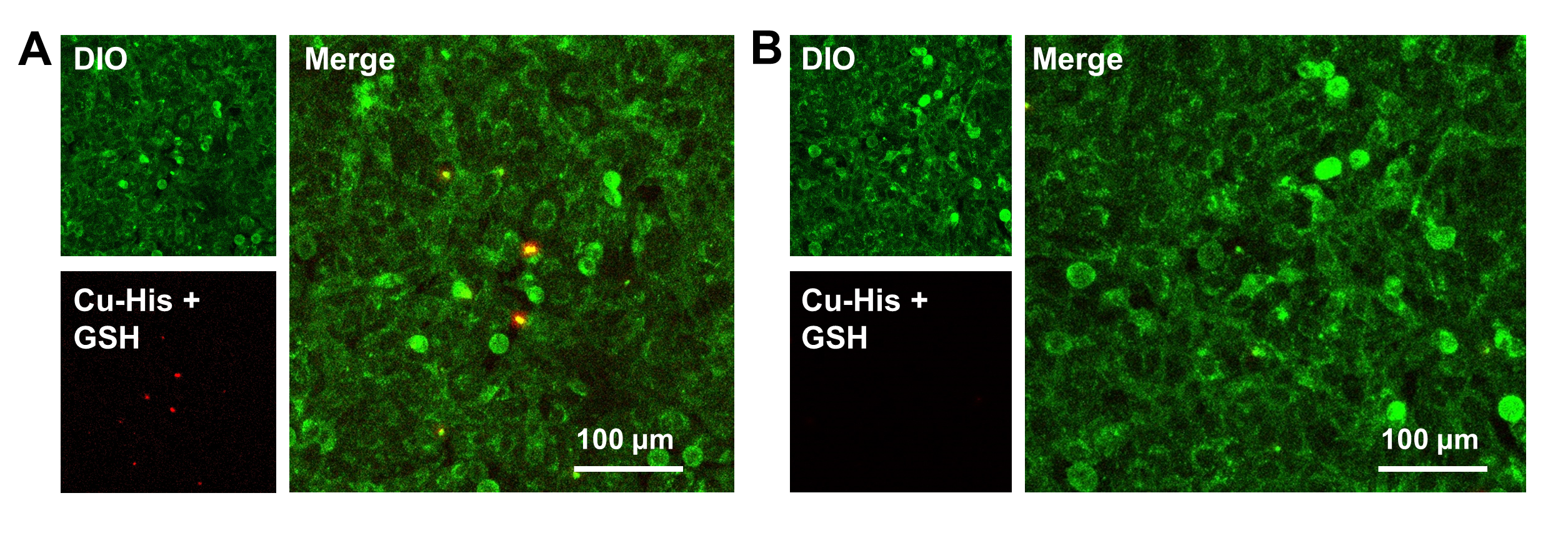
**

**Figure S18.** (A)After 2 h, the reaction between endocytosed Cu-His NPs and intracellular GSH in GL261 cells, induced red fluorescence. Green: DIO. (B) Red fluorescence disappeared after 8 h of co-culture of endocytosed Cu-His NPs with GL261 cells. Green: DIO.


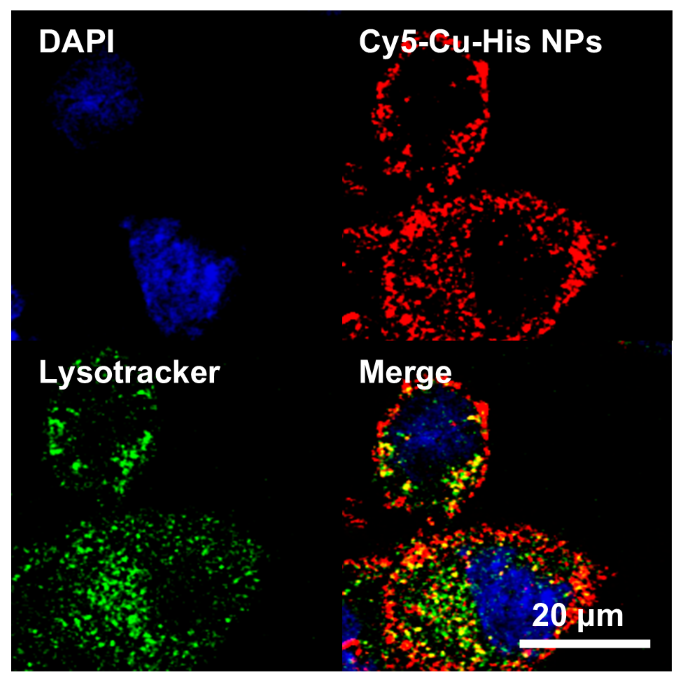


**Figure S19.** CLSM images of GL261 cells in the presence of Cu-His NPs. Blue: DAPI; green: Lysotracker; red: Cu-His NPs labeled with Cy5.


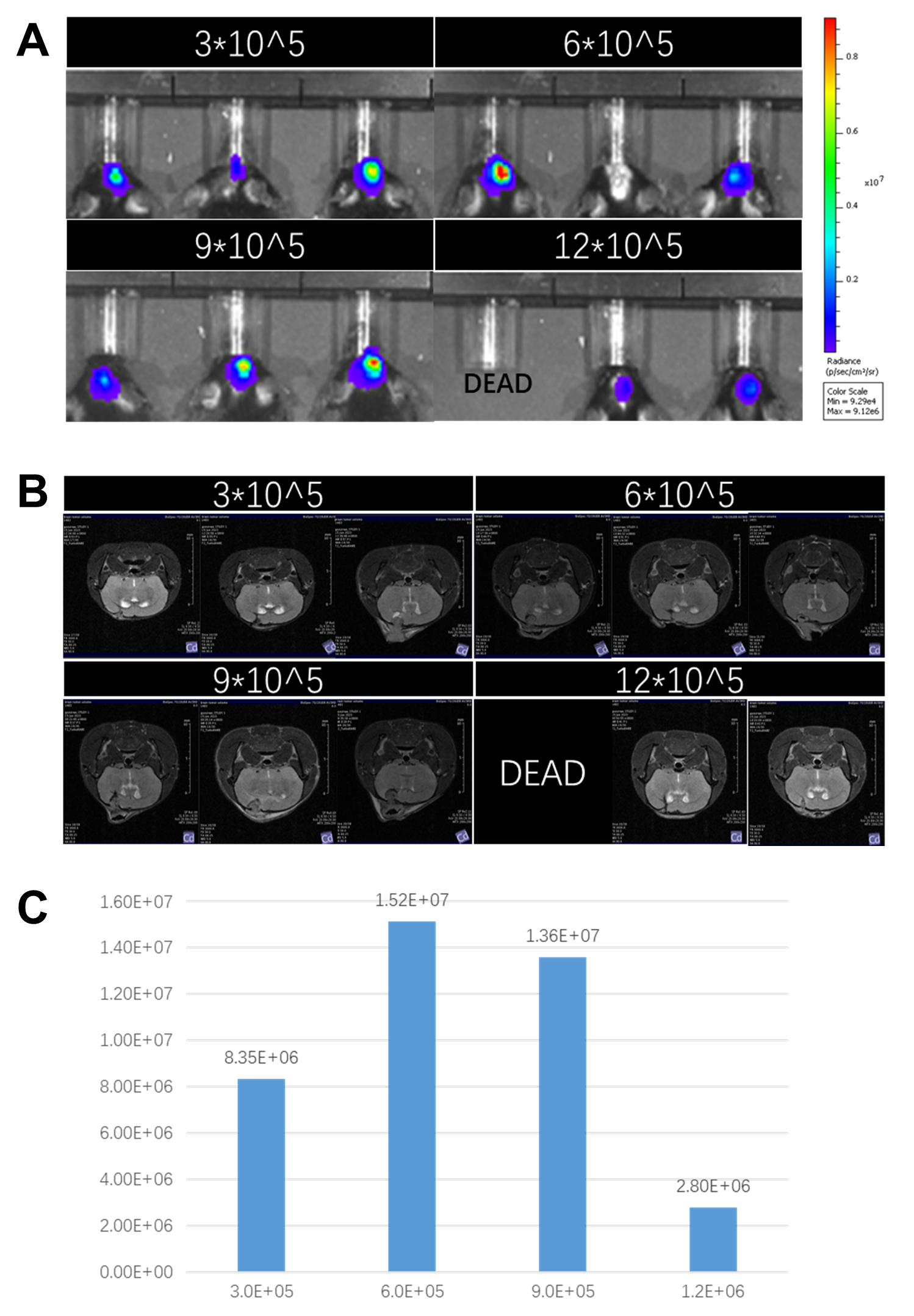


**Figure S20.** (A) In vivo bioluminescence images of implanted Luc-GL261 cells after 7 days (n = 3). (B) T2-weighted MR images of implanted Luc-GL261 cells after 7 days (n = 3). (C) Quantification of bioluminescence imaging of implanted Luc-GL261 cells after 7 days.


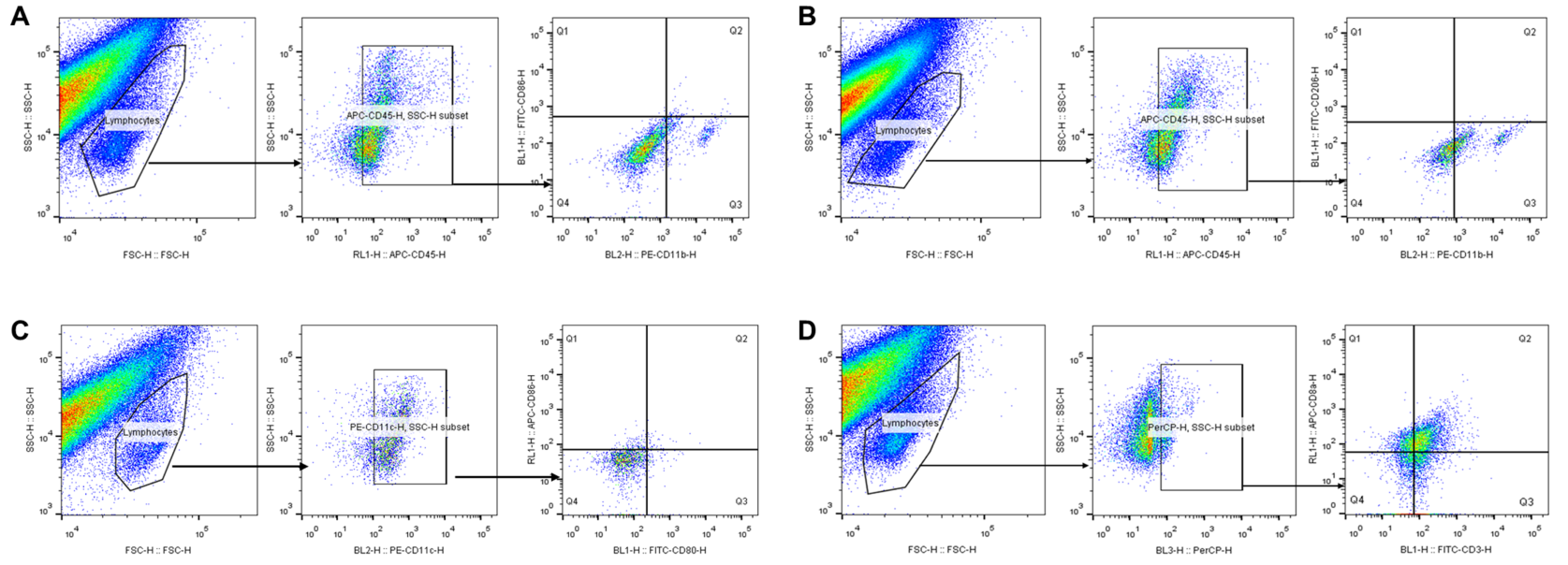


**Figure S21.** Flow Cytometry Gating Strategy of (A) M1-type TAM, (B) M2-type TAM, (C) DC Cells and (D) CD8+ T Cells in Recurrent Tumor Lesions.
